# A comparative analysis of HMGB1 and pCTS-L immunomodulatory properties in human peripheral blood mononuclear cells

**DOI:** 10.3389/fimmu.2026.1764230

**Published:** 2026-03-16

**Authors:** Li Lou, Xiaoling Qiang, Cassie Shu Zhu, Brian Xiong, Weiqiang Chen, Jianhua Li, Kevin J. Tracey, Haichao Wang

**Affiliations:** 1 The Feinstein Institutes for Medical Research, Northwell Health, Manhasset, NY, United States; 2 Departments of Emergency Medicine and/or Molecular Medicine, Donald and Barbara Zucker School of Medicine at Hofstra/Northwell, Hempstead, NY, United States

**Keywords:** chemokines, cytokines, HMGB1, human peripheral blood mononuclear cells, immunomodulatory, inflammasome pathway, inflammatory, inflammatory diseases

## Abstract

High Mobility Group Box 1 (HMGB1) and Procathepsin L (pCTS-L) are crucial inflammatory mediators, yet their immunomodulating properties in human immune cells have not been systematically compared. This study employed RNA-sequencing to comparatively analyze their transcriptional effects on primary human peripheral blood mononuclear cells (PBMCs). Our findings demonstrate that while both mediators elicited significant transcriptional changes indicative of robust inflammatory responses, HMGB1 consistently induced a more extensive and diversified inflammatory program. Specifically, at a lower concentration of 0.5 µg/ml, HMGB1 triggered nearly four times more differentially expressed genes (DEGs) than pCTS-L (2.0 µg/ml). Despite this quantitative difference, an overlap of 412 DEGs (272 upregulated, 140 downregulated) revealed shared core inflammatory pathways, including the extensive upregulation of pro-inflammatory cytokines (e.g., IL1A, IL1B, and IL6), chemokines (e.g., CCL2 and CXCL1), and S100 proteins (e.g., S100A8, S100A9, and S100A12). Both mediators also converged on activating the non-canonical NF-κB pathway, evidenced by NFKB2 and RELB upregulation, suggesting a common underlying regulatory mechanism. Notably, HMGB1 uniquely upregulated CASP4 and CASP5—key components of the non-canonical inflammasome pathway—and a broader spectrum of cytokines and chemokines (e.g., IL23A, CXCL5). These findings delineate the distinct yet overlapping roles of HMGB1 and pCTS-L in orchestrating immune responses, offering a foundation for targeted therapeutic development for inflammatory diseases.

## 1 Introduction

Sepsis, a life-threatening acute inflammatory syndrome, accounts for nearly 20% of deaths worldwide and imposes an annual economic burden exceeding $62 billion in the U.S. alone. In contrast, rheumatoid arthritis (RA) is a chronic autoimmune disease affecting 0.5–1% of the global population (1), characterized by persistent synovial inflammation and progressive joint destruction. Despite their distinct etiologies and clinical manifestations, both conditions converge on a common immunopathological mechanism: the dysregulated release of key pro-inflammatory cytokines [e.g., TNF, interleukin-1 (IL-1), IL-6], damage-associated molecular patterns (DAMPs, such as HMGB1) (2–10), and late-acting proinflammatory mediators (e.g., procathepsin L, pCTS-L) (11).

We and others have established HMGB1 as a pivotal inflammatory mediator in both sepsis (4, 5, 12–14) and RA (6–10). Upon binding to cell surface receptors like Toll-like Receptor 4 (TLR4) (15, 16), extracellular HMGB1 induces various cytokines (e.g., TNF, IL-6, and IL-10) and chemokines (e.g., CXCL5/ENA78, CXCL1/GRO-α, CXCL8/IL-8, CCL2/MCP-1, and CCL7/MCP-3) in human peripheral blood mononuclear cells (PBMCs) (17). At relatively higher concentrations, however, HMGB1 binds the Receptor for Advanced Glycation End products (RAGE) (18), triggering inflammasome activation and macrophage pyroptosis (13, 19).

In contrast, cathepsin L (*Ctsl*) is highly inducible in monocytes/macrophages by bacterial endotoxins and cytokines (e.g., IFN-γ, IL-6, and serum amyloid A, SAA) (20–26). Its precursor, procathepsin L (pCTS-L), can be secreted extracellularly by activated innate immune cells, functioning as a pro-inflammatory mediator in lethal sepsis (25, 27–30) and RA (11). Similar to HMGB1, extracellular pCTS-L can bind both TLR4 and RAGE, inducing multiple cytokines and chemokines in human PBMCs (25). Notably, disruption of TLR4 alone significantly reduced the pCTS-L-induced secretion of most cytokines (e.g., IL-6 and IL-12) and chemokines (e.g., RANTES, MCP-1, MIP-1γ, and LIX), while the complete induction of MIP-2/GRO-β and KC/GRO-α required both TLR4 and RAGE (25). This suggests that, like HMGB1, pCTS-L also employs different receptors to trigger distinct inflammatory signaling pathways in various innate immune cell types (28).

While both pCTSL and HMGB1 are recognized for their roles in promoting inflammation (28), a comprehensive comparison of their distinct and overlapping immunomodulatory gene expression profiles in primary human PBMCs remains underexplored. This study aimed to systematically compare the immunomodulatory properties of recombinant HMGB1 and pCTS-L in human PBMCs using high-throughput RNA-sequencing. By identifying differentially expressed genes (DEGs) and performing Gene Ontology (GO) enrichment and Gene Set Enrichment Analysis (GSEA), we sought to elucidate the common and unique transcriptional programs activated by these two pro-inflammatory mediators, thereby shedding light on their comparative roles in orchestrating immune responses.

## 2 Materials and methods

### 2.1 Human peripheral blood mononuclear cell isolation and culture

Human blood was purchased from the New York Blood Center (NYBC, Long Island City, NY, USA), and PBMCs were isolated by density gradient centrifugation through Ficoll (Ficoll-Paque PLUS) as previously described (25, 31). Due to strict anonymization protocols implemented to protect donor identity and privacy, we were not provided with detailed demographic or clinical information, such as age, sex, or smoking status, for these three individuals (n = 3) who donated blood for PBMC isolation. In accordance with our institutional IRB policy, our requests for de-identified blood samples that lack links to subject identities are not classified as “Human Subject Research” under the Common Rule (45CFR46) or HIPAA (45CFR164). Accordingly, our proposed use of unused/wasted and de-identified human blood or blood products (e.g., leukocytes) from NYBC has been granted IRB Exempt Status. PBMCs were cultured in RPMI-1640 supplemented with 10% human serum, 2 mM L-glutamine, 100 U/mL penicillin, and 100 µg/mL streptomycin (all from Invitrogen). For experiments, PBMCs were stimulated with recombinant human HMGB1 (0.5 µg/ml) or pCTS-L (2.0 µg/ml) for 16 hours, cell-conditioned medium was collected for Cytokine Antibody Array analysis, and total RNAs were harvested for RNA sequencing. Given that transcriptional responses to inflammatory stimuli are dynamic, with some genes being activated rapidly while others require more prolonged signaling to induce expression (25), we deliberately chose an extended 16-hour incubation period. This duration allows sufficient time for transcriptomic changes of both early- and late-acting inflammatory mediators, enabling us to capture a more complete picture of the overall inflammatory and regulatory profile triggered by HMGB1 and pCTS-L.

### 2.2 Cytokine antibody array analysis

Cell-conditioned medium was collected from PBMC cultures after 16 h of stimulation, and the levels of cytokines and chemokines in human PBMC culture supernatants were determined via human Cytokine Antibody Arrays (e.g., Cat. # AAH-CYT-3-8, RayBiotech Inc., Norcross, GA, USA) as previously described (25, 31–33). Arrays were performed according to the manufacturer’s protocol, followed by chemiluminescent detection as previously described (25, 31).

### 2.3 Preparation of recombinant HMGB1 and pCTS-L proteins

Recombinant rat HMGB1, a 33 kDa fusion protein with an N-terminal calmodulin-binding peptide (CBP, 3 kDa) tag, was expressed in *E. coli* BL21 (DE3) pLysS cells after its cDNA was cloned and inserted into a pCAL-n vector as previously described (4, 31, 34). Recombinant CBP-tagged HMGB1 was purified via calmodulin-affinity chromatography, and endotoxins were removed from the HMGB1 preparation via Triton X-114 extraction (34). Briefly, the solution was mixed with 5% (v/v) Triton X-114, incubated for 20 min at room temperature, and then centrifuged (16,000 g, 8 min, room temperature). The resulting HMGB1-containing aqueous phase was collected, and its LPS content was determined using a chromogenic Limulus amebocyte lysate (LAL) QCL-1000 assay (Cambrex Bio Science Walkersville Inc).

The cDNA encoding for human pCTS-L was cloned into a pReceiver expression vector downstream of a T7 promoter with an N-histidine tag, and recombinant pCTS-L protein was expressed in *E. coli* BL21 (DE3) pLysS as previously described (25). The inclusion body-associated recombinant pCTS-L protein was isolated by differential centrifugation and urea solubilization before refolding in Tris buffer (pH 8.0) containing N-lauroylsarcosine. The recombinant pCTS-L protein with N-His Tag was then further purified by histidine-affinity chromatography, followed by extensive Triton X-114 extractions to remove contaminating endotoxins (25). Recombinant HMGB1 and pCTS-L proteins were tested for LPS content by the chromogenic *Limulus* amebocyte lysate assay (Endochrome; Charles River), and the endotoxin content was less than 0.01 U per microgram of recombinant protein.

#### 2.3.1 RNA sequencing and bioinformatics analysis

Total RNAs were isolated from human PBMCs of different donors stimulated with HMGB1 (0.5 µg/ml) or pCTS-L (2.0 µg/ml) using the RNeasy Mini Kit (Qiagen) according to the manufacturer’s instructions. RNA quantity and initial quality were assessed using a NanoDrop spectrophotometer, which required A260/280 and A260/230 ratios both > 1.8. Samples meeting these criteria were then sent to a sequencing facility, where RNA integrity was further confirmed using an Agilent Bioanalyzer. Only samples with an RNA Integrity Number (RIN) of 8 or above were accepted for RNA sequencing. cDNA libraries were prepared using the TruSeq RNA Library Prep Kit v2 (Illumina) and sequenced on an Illumina NovaSeq 6000 platform to generate 100 bp paired-end reads. Raw sequencing reads were quality-checked using FastQC, trimmed with Trimmomatic, and aligned to the human reference genome (GRCh38) using the STAR aligner v.2.5.2b. Gene counts were quantified using StringTie and DESeq2 was used for differential gene expression analysis.

##### 2.3.1.1 Differential gene expression analysis

The DESeq2 package in R software was utilized for identifying differentially expressed genes (DEGs) between the HMGB1- or pCTS-L-treated experimental groups and untreated controls. The Wald test was employed to calculate adjusted *P*-values and log_2_ fold changes. Genes were considered significantly differentially expressed based on an adjusted *P*-value < 0.05 and an absolute log_2_ fold change (|log_2_FC|) ≥ 1.

##### 2.3.1.2 Visualizing DEGs with volcano plots

Volcano plots were generated to visually represent the distribution of DEGs, illustrating both log_2_ fold change and statistical significance. These plots were created using the ggplot2 R package, with significantly upregulated and downregulated genes highlighted in distinct colors.

##### 2.3.1.3 Gene Ontology enrichment analysis

To gain a higher-level, mechanistic understanding of the biological roles and pathways affected by the experimental conditions, Gene Ontology (GO) enrichment analysis was performed. This method moves beyond simply listing individual differentially expressed genes by identifying broader functional categories in terms of their associated biological processes (BP) within the differentially expressed gene (DEG) sets. The analysis was conducted using the clusterProfiler R package. Enrichment terms were considered statistically significant at an adjusted *P*-value < 0.05, determined using Fisher’s exact test. Subsequently, *P*-values were adjusted to account for multiple hypothesis testing, and only terms with an adjusted *P*-value < 0.05 were considered statistically significant.

##### 2.3.1.4 Gene set enrichment analysis

Gene set enrichment analysis (GSEA) was performed to identify significantly enriched pathways using the hallmark gene sets from the Molecular Signatures Database (MSigDB). This method determined whether defined gene sets exhibited statistically significant, concordant differences in gene expression between untreated controls and experimental groups treated with either HMGB1 or pCTS-L. Unlike traditional differential expression analysis, which typically focuses on individual genes surpassing an arbitrary significance threshold, GSEA evaluates the cumulative distribution of all genes within a predefined set, allowing for the detection of even subtle yet coordinated transcriptional changes. The primary objective of this analysis was to identify biological pathways, processes, or molecular functions that are coherently altered by HMGB1 or pCTS-L in human PBMCs.

## 3 Results

### 3.1 Preparation of recombinant HMGB1 and pCTS-L to examine their impact on cytokine/chemokine secretion in human PBMCs

**Figure 1:**
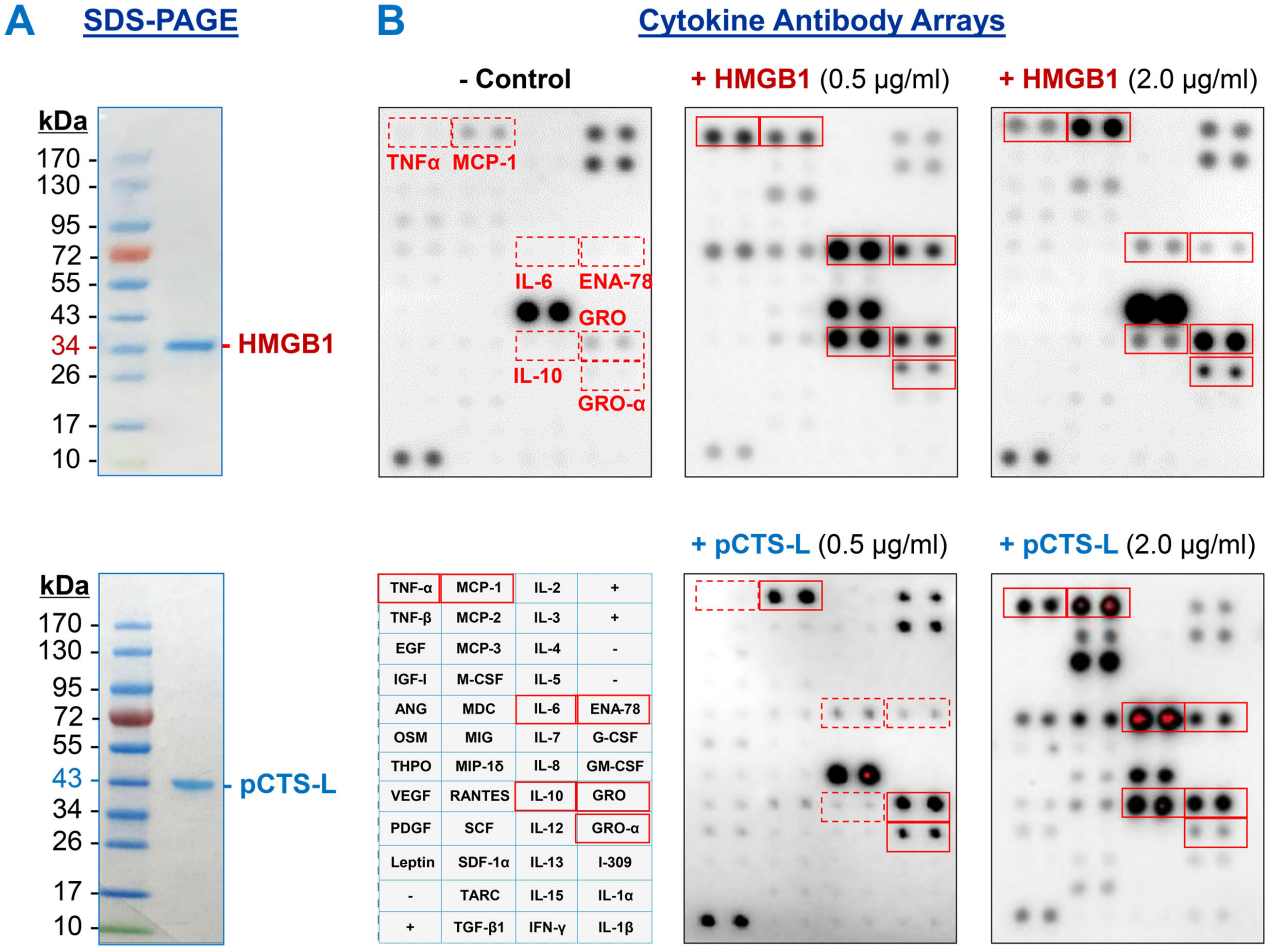
Preparation of recombinant HMGB1 and pCTS-L and their impact on cytokine/chemokine expression in human PBMCs. **(A)** SDS-PAGE analysis of recombinant human HMGB1 (~ 30 kDa) bearing an N-terminal calmodulin-binding peptide (CBP, 3 kDa) tag, and human pCTS-L (~ 40 kDa) containing an N-terminal 6 × Histidine tag (~ 1.0 kDa). **(B)** Cytokine antibody array analysis of HMGB1- and pCTS-L-induced cytokine and chemokine secretion in human PBMCs. At a lower concentration (0.5 µg/ml), HMGB1 stimulated comparable levels of key cytokines (including TNF, IL-6, IL-10, ENA-78, and GRO) to those induced by pCTS-L at a higher concentration (2.0 µg/ml). Consequently, these specific concentrations were chosen to ensure a comparable initial stimulus, thereby enabling a more effective comparison of the downstream transcriptional responses induced by each ligand.

To compare the biological activities of HMGB1 and pCTS-L, recombinant proteins were prepared and characterized by SDS-PAGE analysis (**Figure 1A**). Recombinant HMGB1, bearing an N-terminal calmodulin-binding peptide (CBP) tag, migrated as a distinct band of at approximately 33 kDa, consistent with its predicted molecular weight of 30 kDa for the protein and 3 kDa for the CBP tag. Similarly, recombinant pCTS-L, containing an N-terminal 6 × His tag, was observed as a single band at approximately 41 kDa, matching its expected molecular weight of 40 kDa for the protein and 1.0 kDa for the 6 × His tag. These data confirm that both recombinant HMGB1 and pCTS-L were obtained as highly purified proteins with their predicted molecular weights.

Although literature suggests that the redox state of HMGB1 may affect its proinflammatory activities (35, 36), our recombinant HMGB1 was generated and purified under strictly non-reducing conditions. This approach was specifically chosen to preserve the native disulfide bonds and the physiologically relevant redox state of the protein. Consequently, the cysteine residues in our recombinant HMGB1 are expected to retain their native configuration, including the potential for disulfide bond formation between residues 23 and 45, in contrast to the uniform reduction that occurs with dithiothreitol (DTT) or beta-mercaptoethanol during purification. While explicit biochemical analyses were not performed to determine the precise distribution of disulfide isoforms (e.g., all-reduced, disulfide-bonded, or terminally oxidized), the non-reducing purification conditions suggest that the protein used in our studies would primarily exist as a mixture of redox forms. This composition enables the elicitation of diverse biological responses, rather than exclusively a fully reduced state.

Following characterization, their cytokine/stimulating properties were compared in human PBMCs. Cytokine antibody array analysis revealed marked differences in the ability of HMGB1 and pCTS-L to induce cytokine and chemokine secretion in human PBMCs (**Figure 1B**). Specifically, at a lower concentration (0.5 µg/ml), HMGB1 stimulated comparable levels of key inflammatory mediators, including TNF, IL-6, IL-10, ENA-78, and GRO, to those induced by pCTS-L at a four-fold higher concentration (2.0 µg/ml). This differential potency indicates that HMGB1 is a more potent stimulator of cytokine and chemokine release in human PBMCs compared to pCTS-L. Consequently, these specific concentrations (0.5 µg/ml for HMGB1 and 2.0 µg/ml for pCTS-L) were strategically selected for subsequent experiments to ensure a comparable initial stimulus for both ligands, thereby facilitating a more effective and direct comparison of their downstream transcriptional responses.

### 3.2 Identification of differentially expressed genes

**Figure 2:**
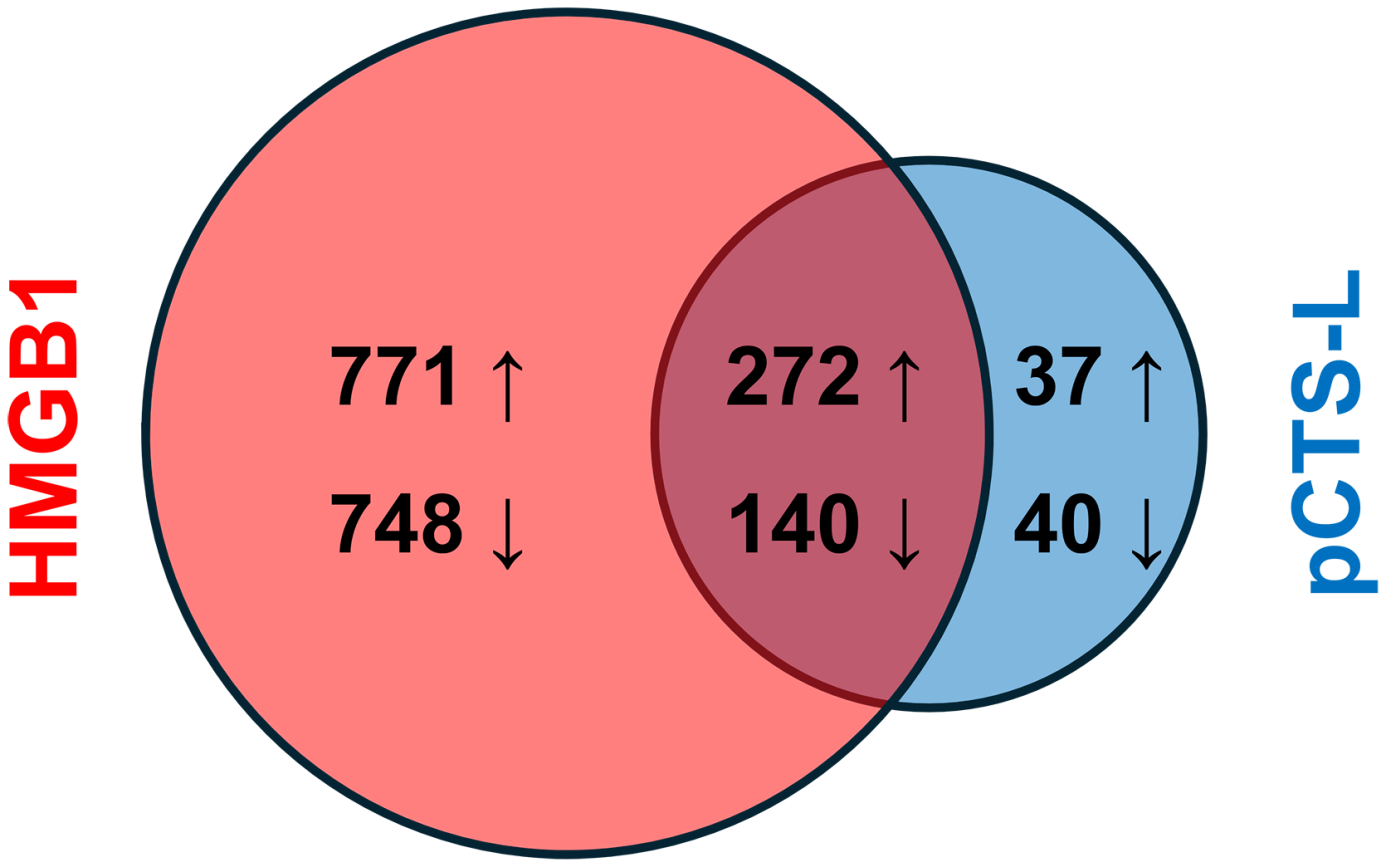
Venn diagram of significant differentially expressed genes. A Venn diagram showing the overlap between pCTSL-induced and HMGB1-induced DEGs.

To compare the immunomodulatory effects of HMGB1 and pCTSL on human PBMCs, we first identified specific genes that were differentially expressed upon treatment with each mediator compared to the untreated control (**Supplementary Material 1**). In HMGB1-treated PBMCs, a total of 1931 significant DEGs were detected, comprising 1043 (771 + 272) upregulated (**Table 1**) and 888 (748 + 140) downregulated genes (**Table 2**, **Figure 2**). In pCTS-L-treated PBMCs, a total of 489 significant DEGs were detected, comprising 309 (272 + 37) upregulated and 180 (140 + 40) downregulated genes (**Figure 2**). This indicates that HMGB1 induces a more extensive alteration in gene expression compared to pCTS-L. However, this DEG count merely reflects the breadth of transcriptional change, and does not necessarily equate to the extent of biological potency.

**Table 1:** Up-regulated genes in HMGB1- or pCTS-L-stimulated human PBMCs.

Stimulus	Genes
HMGB1 (771)	A4GALT, AANAT, AASS, ABCA1, ABCB5, ABCC6, ABHD17C, ABTB2, AC004540.4, AC004980.7, AC005102.1, AC005264.2, AC005301.9, AC005578.3, AC008641.1, AC008984.2, AC010518.2, AC022816.2, AC067945.4, AC079325.6, AC092066.1, AC092484.1, AC108676.1, AC112198.1, ACHE, ACSL3, ACVR1B, ACVRL1, ADAM12, ADAM8, ADARB1, ADGRE2, ADM2, AF165138.7, AFDN, AFF2, AGK, AGRN, AIFM2, AK4P1, AK8, AKT3, ALDH1L2, AMPH, ANKRD1, ANKRD22, ANKRD33B, ANPEP, AOX1, AP000355.2, AP000695.4, APOL1, APOO, APP, AQP3, AQP9, AREG, ARG2, ARL2-SNX15, ARMC12, ARNT2, ARSB, ASNS, ATOH8, ATP10B, ATP13A3, ATP2C1, AVIL, AZIN1, B4GALT1, B4GALT6, BASP1, BATF, BATF3, BCL2L11, BCL6, BCL9L, BHLHE40-AS1, BIRC3, BMP6, BRCA2, BRSK1, BTBD11, BX255923.3, C10orf10, C11orf95, C17orf107, C1orf115, C1orf122, C1orf21, C1RL-AS1, CA12, CA13, CACNA1G, CARMIL3, CASC15, CASP4, CASP5, CATIP, CATIP-AS1, CCDC71L, CCL19, CCL22, CCL23, CCL24, CCL5, CCNA1, CCND3, CCR7, CD14, CD163, CD226, CD40, CD48, CD55, CDKN2D, CEACAM19, CEACAM3, CEMIP, CEP112, CEP135, CEP170B, CFAP58-AS1, CH17-472G23.2, CHAC1, CHDH, CHI3L1, CHI3L2, CHPF, CISH, CITF22-49E9.3, CKAP4, CLCF1, CLEC5A, CLLU1OS, CLSTN3, CMB9-22P13.1, CMPK2, CNGB1, CNIH4, COL24A1, CPD, CRISPLD2, CRLF2, CSF3, CSGALNACT1, CSRNP1, CSTA, CTA-126B4.7, CTA-212A2.1, CTA-293F17.1, CTA-384D8.35, CTA-384D8.36, CTB-114C7.4, CTB-171A8.1, CTB-61M7.2, CTC-312O10.3, CTD-2006K23.1, CTD-2207P18.2, CTD-2319I12.2, CTD-2527I21.9, CTD-2589M5.5, CTD-2600O9.2, CTD-3128G10.6, CTD-3214H19.16, CTSL, CXCL13, CXCL16, CXCL2, CXCL3, CXCL5, CXCL6, CYB5R2, CYP1B1, CYP1B1-AS1, CYP27B1, DBNDD2, DCUN1D3, DENND5A, DESI1, DGAT2, DHCR24, DHCR7, DLGAP1-AS2, DNER, DNAH17, DNAJB5, DNAJB9, DNAJC3, DNAJC3-AS1, DSC2, DSE, DUSP1, DUSP16, DUSP4, DUSP5, DUSP6, DYRK3, EBF1, ECE1, EDNRB, EFNA1, EHF, ELOVL7, EMBP1, ENAH, ENPP4, EREG, ERG, ERN1, ETS1, ETV3, F11R, F5, FADS1, FADS2, FADS3, FAM124A, FAM20A, FAM225A, FAM49A, FAM65C, FASN, FBXO6, FCER1G, FCGR1A, FCGR1B, FCGR1CP, FCGR2A, FCGR3A, FCGR3B, FDPS, FDPSP2, FFAR2, FICD, FJX1, FLJ22447, FLOT1, FLOT2, FNDC3B, FSCN1, FSD1L, FTH1, FTH1P10, FTH1P11, FTH1P16, FTH1P2, FTH1P20, FTH1P23, FTH1P8, FUT7, G0S2, GALNT6, GBP1, GBP1P1, GBP4, GBP5, GCNT4, GGT1, GIPR, GK5, GK-AS1, GK-IT1, GLIS3, GLTPD2, GNA15, GNAI1, GP1BA, GPR132, GPR157, GPR68, GRASP, GRHL1, GRINA, GRK6, GXYLT2, GYPC, H1F0, HAPLN3, HAS1, HCAR2, HECW2, HELZ2, HIP1, HIST1H2BC, HIST1H2BH, HIST1H4H, HIST2H2BE, HIST2H2BF, HIVEP1, HLA-DPA3, HLA-DPB2, HLX, HMSD, HPN, HRH1, HRH2, HSD3BP5, HSPA13, HSPA2, HSPA6, IDO2, IFIT3, IGF2BP1, IGFN1, IL10, IL10RB, IL15RA, IL23A, IL2RA, IL31RA, IL36G, IL6ST, IL7, IL7R, INHBA, INSIG2, INSM1, IRF1, IRF6, IRF7, ISG20, ITGA1, ITGAV, ITGB3, JAG1, JHDM1D-AS1, KANK1, KCCAT211, KCNE5, KCNJ15, KCNN4, KIAA1211, KIAA1644, KIAA1958, KIF21A, KL, KLF9, KLHDC7B, KREMEN1, KSR1, LAD1, LAMC1, LAMC2, LAMP3, LAP3, LDLR, LHFP, LILRA1, LILRA5, LILRB2, LILRB4, LINC00158, LINC00243, LINC00528, LINC00563, LINC00622, LINC00623, LINC00877, LINC00884, LINC01093, LINC01094, LINC01095, LINC01137, LINC01268, LINC01358, LINC01482, LINC01619, LIPM, LIPN, LMOD3, LPAR1, LPAR3, LPAR4, LPP-AS2, LRP12, LRP2BP, LRP4, LRP8, LRRFIP1P1, LRRK2, LTBP2, LUCAT1, LYSMD2, MAGEF1, MAGI2-AS3, MAMLD1, MAOA, MAPRE3, MARCHF3, MARCHF9, MARCKSL1, MARCO, MBOAT2, MBOAT7, MCEMP1, MCL1, MCTP2, MEFV, MEI1, MERTK, Metazoa_SRP, MFSD2A, MGAM, MICA, MICALL1, MIR22HG, MIR8061, MMP25, MMP7, MOB3B, MPZL1, MS4A14, MSANTD3-TMEFF1, MSC, MSC-AS1, MSMO1, MT1A, MT-TV, MURC, MVD, MVP, MXD1, MYEOV, MYO1C, N4BP1, NAB1, NAP1L5, NBPF9, NCF1B, NDFIP2, NDP, NDST2, NECTIN4, NFAT5, NFE2L1, NID2, NINJ1, NKG7, NLRC5, NMNAT2, NOD2, NOL3, NOTCH3, NOTCH4, NRN1, NT5DC2, NT5E, NTSR1, NUP62CL, OASL, ODF3B, OLFML2B, OTUD1, OTUD7A, OTUD7B, P2RY2, P2RY6, P4HA2, PANX1, PANX2, PARP10, PARP9, PCDHGB7, PCGF2, PCNX1, PCOLCE2, PDE4A, PDE4D, PDLIM4, PDLIM7, PDP2, PERM1, PFKFB3, PHLDA2, PHLDB1, PID1, PIF1, PILRA, PIM3, PKN3, PLAC8, PLCB4, PLEK2, PLEKHG2, PLEKHN1, PLP2, PLSCR1, PLXNB1, PMEPA1, PNMA1, PNP, PNPLA1, PNRC1, POU6F1, PPBP, PPIF, PPM1N, PPP1R17, PPP1R3B, PPP3CC, PRDM8, PRELID2P1, PRKCDBP, PROB1, PROCR, PRR16, PRRG1, PRRT2, PSD3, PSMA6, PSME2, PSME2P2, PTGER2, PTPN1, PTPN12, PTPN2, PTPRD, PTPRJ, PVR, PXK, QSOX1, RAB13, RAB24, RAB3IP, RAI14, RAP1B, RARA-AS1, RASGRP1, RASL10A, RBMS1P1, RBP5, RCN1, RETN, RFTN1, RFX8, RHBDD2, RHOU, RMDN2-AS1, RN7SKP26, RN7SL124P, RN7SL368P, RNASE2, RNF145, RNF175, RNU4ATAC18P, RP1-102K2.9, RP1-151F17.1, RP1-151F17.2, RP1-232L22:B.1, RP1-28O10.1, RP1-68D18.2, RP1-68D18.4, RP1-93H18.1, RP11-10J5.1, RP11-102N12.3, RP11-1149O23.2, RP11-12A2.1, RP11-1334A24.5, RP11-153M7.3, RP11-17G12.3, RP11-185E8.2, RP11-196G18.3, RP11-203E8.1, RP11-242C19.2, RP11-273B20.1, RP11-274E7.2, RP11-283G6.3, RP11-283G6.4, RP11-317P15.4, RP11-324I22.3, RP11-325F22.2, RP11-329N15.3, RP11-34E5.4, RP11-351I24.1, RP11-353N4.5, RP11-367G18.1, RP11-373D23.3, RP11-37B2.1, RP11-384O8.1, RP11-404F10.2, RP11-415J8.3, RP11-422P24.10, RP11-422P24.12, RP11-439A17.9, RP11-443B7.1, RP11-443B7.3, RP11-44K6.2, RP11-44K6.4, RP11-452H21.4, RP11-454E5.4, RP11-470B22.1, RP11-47I22.2, RP11-48B3.3, RP11-48B3.4, RP11-499E18.1, RP11-512N21.3, RP11-561O23.5, RP11-563J2.3, RP11-571M6.17, RP11-58K22.4, RP11-58K22.5, RP11-612B6.2, RP11-626A5.2, RP11-631N16.2, RP11-631N16.4, RP11-797A18.6, RP11-79H23.3, RP11-800A3.4, RP11-810P12.7, RP11-81A22.4, RP11-81H14.1, RP11-8H2.1, RP13-314C10.5, RP3-342P20.2, RP4-530I15.9, RP4-607I7.1, RP4-647J21.1, RP4-756G23.5, RPGR, RUBCNL, RUSC1-AS1, SAMD14, SAMSN1, SAMSN1-AS1, SAT1, SBNO2, SC5D, SCARF1, SCG3, SCN1B, SDC4, SELK, SELM, SEMA4B, SEMA4C, SEMA4D, SERPINB2, SERPINB9, SESN2, SH2D3A, SHROOM3, SHROOM4, SIGLEC5, SIK3, SLAMF1, SLC11A1, SLC16A6, SLC22A4, SLC24A3, SLC25A23, SLC25A37, SLC30A1, SLC43A2, SLC4A7, SLC5A3, SLC5A9, SLC7A11, SLC7A11-AS1, SLC7A5, SLC9A7P1, SLCO4A1, SLFN5, SMCO2, SMOX, SNX25, SOCS1, SOCS2, SORCS2, SOX5, SPACA6P-AS, SPAG1, SPATA20P1, SPHK1, SPRED2, SPTBN5, SQLE, SQRDL, SREBF1, SRGN, SRSF12, SSC4D, SSPN, ST3GAL2, ST6GALNAC3, STAT4, STK17A, STK17B, STK26, STK33, STRIP2, STX1A, STXBP5-AS1, SUSD6, SYN1, SYNPO2, TBC1D30, TBK1, TCEAL9, TCTEX1D1, TFRC, TGFBI, THAP10, THBD, THBS1, TIMP1, TLCD2, TLR2, TM7SF2, TMCC2, TMEM132A, TMEM139, TMEM150B, TMEM158, TMEM176A, TMEM176B, TMEM26, TMEM35B, TMEM41B, TMEM45B, TMEM92, TMEM92-AS1, TNC, TNF, TNFRSF10A, TNFRSF18, TNIP2, TPST1, TRAF3IP2, TREM1, TRIM6, TRIP10, TRPA1, TSC22D3, TSFM, TTC39A, TUBA1C, TUBE1, TXN, TYMP, UBALD2, UBE2D1, ULBP1, UPB1, USP30-AS1, VAV1, VCAN, VDR, VMO1, VN1R105P, VNN1, VNN3, VRK2, VSIG10L, WASF1, WDR63, WFDC21P, WI2-1896O14.1, WIPI1, WNK2, XK, ZBED2, ZBED6CL, ZBTB17, ZHX2, ZMIZ2, ZNF259P1, ZNF319, ZNF462, ZNF697, ZSWIM8-AS1
pCTS-L (37)	AKR1B1, ANOS1, BMT2, CCL3L3, CH25H, CMKLR1, CPAMD8, CPSF1P1, CTNND2, DHRS4L2, FAS, FZD7, GPR141, HDGF, IRAK3, KCNJ10, MPP7, NID1, OLIG1, PANK1, PLAU, PLTP, PTX3, RASSF4, RDX, RFPL2, RP11-629O1.2, RP11-707P17.2, SEMA3A, SMARCD3, TBC1D16, TBC1D17, TMEM97, TOR3A, UGCG, VOPP1, ZC3H12C
Both HMGB1 and pCTS-L (272)	ABCG1, AC010980.2, AC013444.1, AC061992.2, ACO1, ACOD1, ACP2, ACSL1, ACSL4, ADA, ADAMDEC1, ADAMTS14, ADGRE1, ADM, ADORA2A, ADORA2A-AS1, AHR, AK4, AMPD3, ANKLE1, AP001063.1, AP001189.4, AP001469.5, APOL3, ARID5B, ARNTL2, ASAP2, B4GALT5, BCL3, BHLHE41, BTG3, C15orf48, C17orf96, C1QTNF1, C1orf61, C1RL, C1S, C3, C6orf223, CCL18, CCL2, CCL20, CCL3, CCL4, CCL4L2, CCL7, CCL8, CCL20, CCM2L, CD274, CD300E, CD38, CD44, CD80, CD82, CDC42EP2, CFB, CFP, CHST2, CLEC4D, CLEC4E, CLEC6A, CLIC4, CLU, CRIM1, CSF2RB, CTD-2527I21.14, CTD-2616J11.3, CTD-3128G10.7, CTTN, CXCL1, CXCL8, DDIT4, DLEU7, DLL1, DMXL2, DOCK4, DRAM1, DTX2, EBI3, EHD1, ENPP2, EPB41L3, ETS2, ETV5, FAM60A, FCAR, FILIP1L, FMN1, FOSL2, FPR1, FPR2, FTH1P7, GATSL3, GCAT, GCH1, GGH, GJA3, GJB2, GK, GLT1D1, GPR84, GRAMD1A, GUCY1B2, HCK, HEY1, HIF1A, HMGN2P46, HMMR, HS3ST3B1, HSD11B1, ICAM1, IDO1, IER3, IL1A, IL1B, IL4I1, IL6, IL18, IRAK2, ITGB8, ITPR2, JAK3, KB-1507C5.4, KIFC3, KMO, KYNU, LAMB3, LIF, LILRB1, LIMK2, LINC00937, LLNLR-271C9.1, LRRC32, LSS, LY75, LYN, MAP1LC3A, MARCKS, MB21D2, MCOLN2, MCTP1, MDM1, MET, MIR3142HG, MIR3945HG, MMP14, MMP9, MN1, MS4A7, MSANTD3, MT1D, MT1DP, MT1E, MT1F, MT1G, MT1H, MT1JP, MT1L, MT1M, MT1X, MT2A, MTF1, MTRNR2L8, MYH11, MYO1G, N4BP1, NAB1, NAMPT, NAMPTP1, NCF1, NCF1C, NECTIN2, NFKB1, NFKB2, NFKBIA, NFKBIZ, NFIL3, NOL4L, NR1H3, OLIG2, OR7E140P, ORM1, OSM, PDE4B, PDPN, PLAGL2, PLAUR, PLD1, PLEKHG3, PLEKHM3, PLK3, PLXNA2, PNKD, PPA1, PSTPIP2, PTGER4, PTGIR, PTGS2, QPCT, RELB, RGS16, RHBDF2, RILPL2, RIMS3, RNF144B, RNF19B, RP1-310O13.7, RP11-157E21.1, RP11-211G3.2, RP11-212I21.2, RP11-212I21.5, RP11-214O1.2, RP11-465L10.10, RP11-519G16.3, RP11-519G16.5, RP11-624C23.1, RP11-662I13.2, RP11-81H14.2, RP13-452N2.1, RP3-393E18.2, S100A12, S100A8, S100A9, SEMA4A, SERPINA1, SGPP2, SH3PXD2B, SIGLEC10, SKIL, SLAMF7, SLC16A10, SLC1A2, SLC2A3, SLC2A6, SLC30A4, SLC39A8, SLC41A2, SPACA6, SPATC1, SMPD5, SMPDL3A, SNX10, SOCS3, SOD2, ST3GAL1, STEAP3, STOM, SYT17, TBC1D9, TNFAIP3, TNFAIP6, TNIK, TNIP1, TNIP3, TNFRSF4, TNFRSF9, TNFSF9, TP53INP2, TRAF1, TXLNB, UPK3B, USP13, VEGFA, VILL, WNT5A, WTAP, ZB TB10, ZC3H12A, ZP3, ZSWIM4

**Table 2:** Down-regulated genes in HMGB1- or pCTS-L-stimulated human PBMCs.

Stimulus	Genes
HMGB1 (748)	A2M-AS1, AATBC, ABAT, ABCB9, ABHD14A, ABI3, AC004540.5, AC004988.1, AC006116.17, AC006116.22, AC007246.3, AC007386.2, AC011899.9, AC015688.3, AC079767.4, AC093627.8, AC093673.5, AC104532.4, AC104653.1, ACOT1, ACOT2, ACOT4, ACSM3, ACSM5, ACSS1, AD000684.2, ADAMTS10, ADAMTS6, ADCY3, ADD2, ADGRA3, ADGRG6, ADHFE1, ADORA3, ADRA2B, ADRB2, ADSSL1, AF127936.5, AFF3, AIG1, AKAP6, ALDH1A1, ALDH5A1, ALDH7A1, ALK, ALS2CL, AMDHD2, AMIGO1, AMOTL1, ANGPT1, ANK1, ANK3, ANKRD29, ANTXR1, ANXA11, AP001055.6, AP001257.1, AP003774.4, APBA1, APOC1, APOE, ARHGAP15, ARHGEF28, ARL4C, ARMC2, ASGR1, ASGR2, ASPHD1, ASRGL1, ATP1B4, AZU1, B3GNT8, B9D1, BACE1, BAIAP2, BAIAP2-AS1, BBOF1, BCAS1, BEND3P2, BGN, BMF, BMP2, BNC2, BTF3P7, BUB1B, C10orf128, C11orf21, C11orf45, C11orf65, C15orf52, C16orf47, C16orf54, C17orf100, C1orf127, C1QB, C1QC, C20orf27, C21orf62-AS1, C2CD2, C4orf36, C5orf66, C9orf106, CA3-AS1, CABP4, CACNA2D3, CADM1, CALCR, CALD1, CAMK1D, CAMKK1, CAMP, CAV1, CBFA2T3, CBR3-AS1, CBS, CCDC102B, CCDC153, CCDC34, CCDC78, CCNB2, CCSER1, CD101, CD1A, CD1C, CD200R1, CD22, CD244, CD300A, CD302, CD36, CD4, CD72, CD74, CD84, CDC20, CDC42EP3, CDCA7L, CDH11, CDH2, CDH26, CDKN2B, CDR2, CEBPA, CEBPA-AS1, CECR1, CELSR2, CENPA, CENPF, CENPU, CENPV, CENPW, CERKL, CFD, CFH, CHCHD6, CHIT1, CHPT1, CIITA, CLCN1, CLEC10A, CLEC12A, CLEC12B, CLEC1B, CLEC4F, CLECL1, CLMP, CLYBL, CMBL, CNN2P9, COL12A1, COL13A1, COL16A1, COL18A1, COL1A1, COL1A2, COL22A1, COL3A1, COL5A1, COL5A2, COL6A3, CPVL, CRHBP, CROCC2, CRYBB1, CST3, CTB-52I2.4, CTC-205M6.1, CTC-251D13.1, CTC-301O7.4, CTC-591M7.1, CTD-2003C8.2, CTD-2006C1.2, CTD-2201I18.1, CTD-2284J15.1, CTD-2531D15.4, CTD-3195I5.4, CTDSPL, CTHRC1, CTSF, CTSK, CTSV, CTTNBP2, CUX2, CX3CR1, CXCR2, CXCR2P1, CYBRD1, CYGB, CYP2U1, CYSLTR1, CYTL1, DACH1, DBP, DCDC2, DCLK2, DCN, DDAH1, DDIAS, DDIT4L, DENND4C, DEPTOR, DHRS11, DIP2C, DISP2, DNASE2B, DNM3, DNMT3A, DOC2A, DOCK3, DOPEY2, DPEP3, DPF3, DST, DTNA, DUS2, DUX4L50, DYRK2, DYRK4, EFNB1, EPDR1, EPHB1, EPHX1, ETNK2, EXTL2, F13A1, F2RL1, FABP5P7, FAIM, FAM109A, FAM117B, FAM151A, FAM19A3, FAM212A, FAM213B, FAM32BP, FAM46A, FAM47E, FAM81A, FAP, FAT1, FAT2, FBLIM1, FBLN5, FBN2, FBXO15, FCGBP, FCMR, FCN1, FDX1, FECH, FES, FFAR4, FGD2, FGD6, FGF9, FGL2, FLJ21408, FN1, FNDC10, FOXM1, FOXRED2, FRMD3, FRMD4B, FRRS1, FTCDNL1, FUCA1, FXYD6, GALNT12, GAPT, GAS2L3, GATM, GCHFR, GCLC, GDF15, GGTA1P, GIMAP7, GINS1, GJA1, GLYATL1P1, GMPR, GNG12, GNG7, GNGT2, GPD1, GPLD1, GPNMB, GPR155, GPR161, GPR176, GRAMD4, GSG2, GSTM2, GUCY1A3, GUSB, HACD1, HADH, HAGHL, HAMP, HAVCR2, HDAC9, HGF, HLA-DMB, HLA-DOA, HLA-DPA1, HLA-DPB1, HLA-DQA1, HLA-DQB2, HLA-DRA, HLA-DRB1, HMOX1, HPCAL1, HPGDS, HS3ST2, HSD17B14, HSF4, HSPE1P18, HSPG2, HTRA4, IER5L, IFITM10, IFNLR1, IGFBP3, IGFBP5, IGKV3-11, IGSF10, IGSF22, IL11RA, IL16, IL18BP, IL1RL2, IL24, INPP4B, INTU, IPCEF1, IQCH-AS1, IQCK, IQGAP2, ITGA11, ITGA3, ITGA4, ITGA9, ITGA9-AS1, ITGAE, ITGB5, ITPKB, IVD, JAKMIP2, JAKMIP3, JAML, JPH4, KANSL1-AS1, KCNA10, KCNA2, KCNAB1, KCNAB2, KCNC4, KCNJ1, KCNJ5, KCNMB4, KEL, KIAA0930, KIAA1211L, KIAA1456, KLF2, KLF3-AS1, KLHDC8B, KLHL13, KLHL33, LAMA4, LDLRAD4, LEP, LGALS2, LGALS3, LGALS9, LGMN, LINC00607, LINC00677, LINC00847, LINC00865, LINC00886, LINC01010, LINC01160, LINC01503, LINC01504, LINC01506, LINC01526, LOX, LOXL2, LPAR6, LRFN4, LRRC75A, LSR, LTA4H, LTB4R, LTBP1, LTBP3, LUM, LY86, LY86-AS1, LY9, LYNX1, M1AP, MACROD1, MAP2K6, MARVELD1, MAST4, MBOAT1, MBP, MEF2C, MELTF, METTL7A, MFI2-AS1, MGAT1, MIR4477B, MITF, MLC1, MLXIPL, MMP2, MPP7, MPPED2, MPZL2, MRC2, MRPS18AP1, MS4A4E, MS4A6A, MS4A6E, MTARC-2, MTCL1, MTURN, MTUS1, MXI1, MYB, MYO1E, MYOZ1, NCAPH, NCOA7, NENF, NFIA, NHSL2, NKAPP1, NLN, NOV, NR1D1, NREP, NRSN2-AS1, NT5DC4, NTAN1, NUPR1, NYNRIN, OAS1, OR52K3P, OSBPL1A, OSBPL5, OSBPL7, OSMR, OTOA, P2RX1, P2RY11, P2RY8, PABPC4, PADI2, PALLD, PAQR8, PAX8, PCSK6, PDE3B, PDGFB, PDGFC, PDK2, PEBP1, PER3, PFKFB2, PGBD5, PGM5, PHACTR1, PHOSPHO1, PHYH, PIK3IP1, PKD2L1, PKP2, PLA2G15, PLAC9, PLCB2, PLCXD1, PLD4, PLEKHG4, PLOD2, PLXDC2, POLQ, POSTN, PPGARG, PPARGC1A, PPARGC1B, PPM1E, PPM1J, PPP2R2B, PPP5D1, PRICKLE1, PRKACB, PRKX, PRR5, PRR5L, PRRX1, PRSS23, PSCA, PTCRA, PTK7, PXDN, QPRT, RAB3IL1, RAB40B, RAB42, RAD51AP1, RAP2B, RARRES1, RASAL1, RASGRF1, RASL11A, RASL11B, RAVER2, RBM11, RCVRN, REPS2, RGS12, RGS18, RHOBTB1, RHPN2, RNF144A, RP1-193H18.2, RP1-27K12.4, RP1-80N2.2, RP11-106M3.2, RP11-1081L13.4, RP11-133K1.12, RP11-142C4.6, RP11-14I17.3, RP11-156K13.3, RP11-15A1.3, RP11-213G2.1, RP11-290F5.2, RP11-327E2.5, RP11-32B5.1, RP11-330A16.1, RP11-334C17.5, RP11-345J18.2, RP11-346C20.3, RP11-346C20.4, RP11-356M20.1, RP11-363E6.3, RP11-368J21.3, RP11-373N22.3, RP11-379F4.4, RP11-395L14.18, RP11-404F10.6, RP11-405M12.4, RP11-426C22.5, RP11-436D10.3, RP11-442O1.3, RP11-456K23.1, RP11-459I19.1, RP11-513M16.7, RP11-513M16.8, RP11-51F16.1, RP11-528G1.2, RP11-529E10.6, RP11-532F12.5, RP11-557H15.3, RP11-564A8.8, RP11-568K15.1, RP11-588K22.2, RP11-598F7.3, RP11-59D5:B.2, RP11-645C24.5, RP11-728F11.4, RP11-73K9.2, RP11-74E22.3, RP11-75C10.6, RP11-762L8.6, RP11-787I22.3, RP11-830F9.5, RP11-84A19.3, RP11-862L9.2, RP11-876N24.3, RP11-876N24.4, RP11-876N24.5, RP11-960L18.1, RP11-96D1.7, RP3-395M20.8, RP4-644L1.2, RP4-657E11.10, RP4-671O14.5, RPL7AP64, RPS6KA2-IT1, RPS6KA4, RRAGD, RRN3P1, RUFY4, S100Z, SAMD13, SAMD4A, SARM1, SCARB1, SCIN, SDC3, SDS, SELL, SENCR, SESN1, SETDB2, SEZ6L, SGK1, SGSH, SHB, SHMT1, SIGIRR, SIGLEC15, SIGLEC17P, SIT1, SLAMF6, SLC18B1, SLC19A1, SLC29A3, SLC2A5, SLC2A9, SLC37A2, SLC40A1, SLC45A3, SLC46A1, SLC6A16, SLCO4C1, SLIT2, SMAD3, SMIM2-AS1, SMPD3, SNRPGP14, SNX24, SNX29, SORBS3, SORL1, SORT1, SOX13, SPACA9, SPAG8, SPARC, SPATA12, SPIRE2, SPN, SPOCK1, SPON1, SRD5A3, SRGAP3, ST3GAL5, ST3GAL5-AS1, ST3GAL6-AS1, ST6GAL1, STARD13, STARD4-AS1, STC1, STC2, STK39, STMN1, STON2, STUM, SULT1B1, SULT1C2, SYN2, SYTL3, TANC2, TBC1D10C, TBC1D4, TCN2, TENM4, TESC, TESK1, TEX2, TIMP3, TIMP4, TLR5, TLR7, TM4SF19, TM4SF19-AS1, TM4SF19-TCTEX1D2, TMEM117, TMEM254-AS1, TMEM86A, TMIGD3, TNFSF12, TNK2-AS1, TNNI2, TNS1, TPM2, TPPP3, TRERF1, TRIM45, TRPV6, TSPAN12, TSPAN4, TXNDC16, TXNRD3, U62631.5, UBXN11, UNC5B, UNC80, UPK3A, USP2, UST, VAT1, VEGFB, VSIG2, VWF, WDR91, WIPF3, WWC1, XCR1, XYLT1, Y_RNA, YAP1, YPEL4, ZBED3-AS1, ZBTB12, ZDHHC1, ZFP36L2, ZNF395, ZNF491, ZNF589, ZNF618, ZNF711, ZSCAN16-AS1
pCTS-L (40)	ACE, ADIRF-AS1, AOC1, APOBEC3A, C5AR2, CAMK1, CD1B, CD1E, CD276, COLEC12, CRABP2, CYSLTR2, EGR2, FAIM2, FAM20C, FCER2, FCGRT, FRMD4A, GPAT3, GPX3, ID2, IFITM3, ITGB7, KLF10, LYZ, MGST2, PDK4, PHLDA1, PLD3, PTGFRN, RAB7B, RGCC, RGS2, RNF122, SEL1L3, SFMBT2, SH3BGRL3, SH3RF3, SLAMF9, TXNIP
Both HMGB1 and pCTS-L (140)	A2M, ABCC5, ABCG2, AC007556.3, AC093627.10, AC093627.9, AC104809.4, ACP5, ADGRD1, ADORA2B, AFAP1L1, AMDHD1, ANKH, ARMC9, ATF3, ATP10A, ATP1B1, ATP6V0D2, C1orf162, C1orf228, C5, CABLES1, CARD14, CAT, CD109, CD180, CD1D, CD300LB, CD52, CD9, CHST13, CLN6, CORO2A, CTD-2135D7.5, DHRS9, DIRAS1, DOK2, DPEP2, EEPD1, EFCAB2, EMP1, EPAS1, EPB41L1, EPHB2, EPHB6, EPS8, EVL, FABP3, FABP4, FABP5, FAM131B, FAM13A, FAXDC2, FBP1, FCGR2B, FHL1, FOLR2, GALM, GOLGA7B, GPR34, GPRC5B, HCST, HHEX, HLA-DMA, HSD3B7, KCNH3, KCNQ1, KCP, KLF4, L3MBTL4-AS1, LIPA, LPL, LSP1, MAML3, MATK, ME3, MEGF6, MIR503HG, MLPH, MNDA, MSR1, MXD4, NLRP1, NRGN, PLCB1, PLIN2, PMFBP1, PNPLA7, PRSS36, PTPRO, RAB11FIP4, RCBTB2, RET, RHOBTB2, RNASE6, RNF125, RP11-1072A3.3, RP11-309L24.4, RP11-344B5.2, RP11-356N1.2, RP11-4B16.4, RP11-527N22.1, RP11-90P13.1, RPH3A, RPS6KA2, RTN4R, SARDH, SEMA3G, SEPP1, SERINC2, SLC22A5, SLC26A11, SLC38A6, SLC39A10, SLC47A1, SLC7A8, SNAI3, SNED1, SPOCD1, SPRY2, SPTBN1, SPTBN4, ST14, ST5, SUCNR1, SULF2, SYNGR1, TESK2, THRA, TLN2, TMEM255A, TMEM37, TMEM91, TNFRSF11A, TNFSF8, TREM2, TREML1, TSPAN32, ZBED3, ZNF703

**Figure 3:**
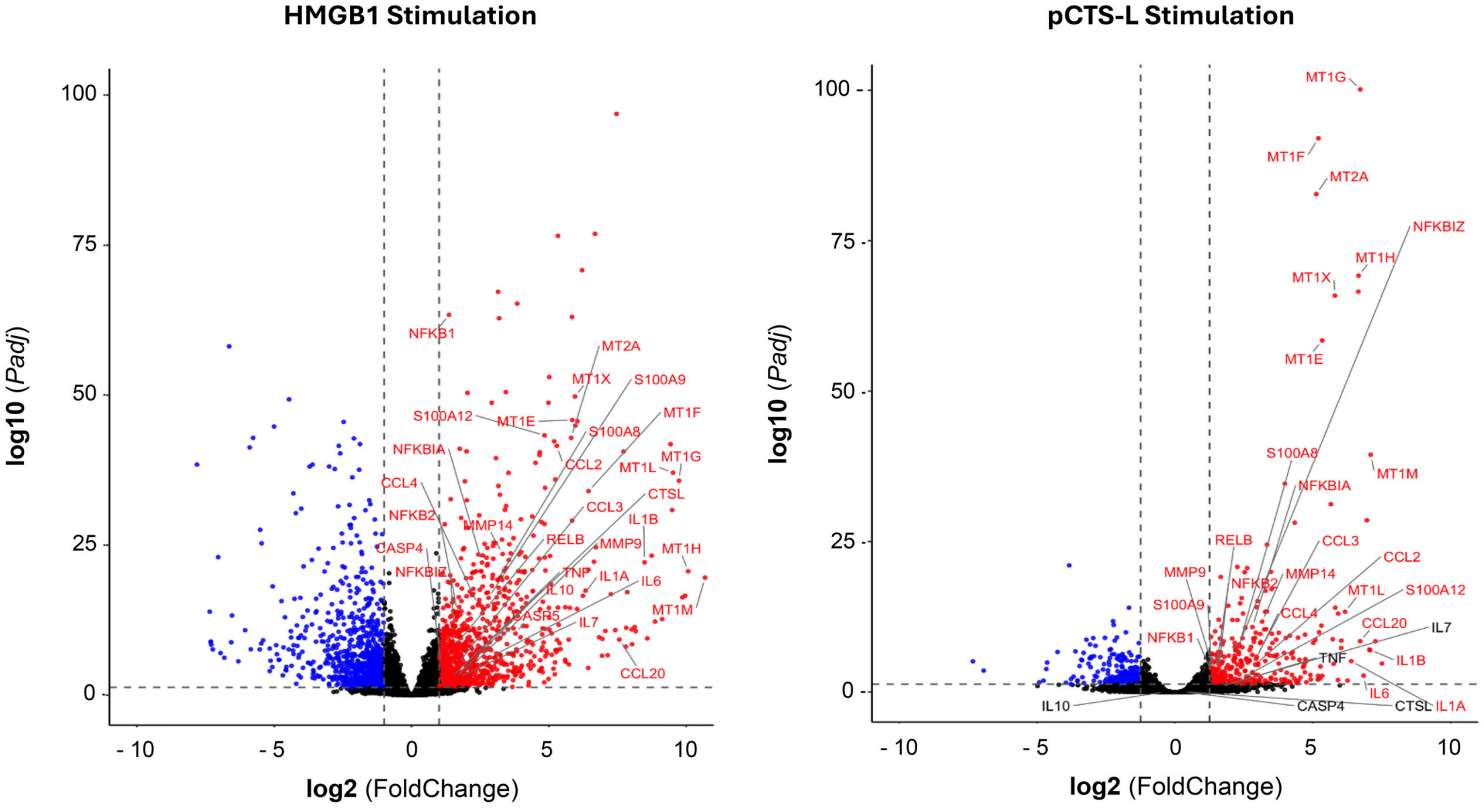
Volcano plot of differentially expressed genes in HMGB1- and pCTSL-stimulated PBMCs. Volcano plots showing upregulated and downregulated genes in HMGB1- or pCTSL-stimulated cells compared to control.

Using online software Venn 2.1, we identified 412 significant DEGs (272 upregulated + 140 downregulated) commonly regulated across both HMGB1- and pCTS-L-stimulated PBMCs (**Figure 2**, **Tables 1**, **2**). This substantial overlap suggests shared inflammatory mechanisms, while the larger number of HMGB1-specific DEGs points to additional or amplified pathways. Volcano plots were generated to visualize the global changes in gene expression for treatment with each mediator, illustrating the magnitude of differential expression against statistical significance (**Figure 3**).

Analysis of the DEGs revealed upregulation of several groups of inflammatory mediators:

#### 3.2.1 Cytokines and chemokines

Both HMGB1and pCTS-L stimulation led to the upregulation of key cytokine-related genes including *IL1A, IL1B, IL4I1, IL6, OSM, IFNA, IFNB*, and *IFNG*. Similarly, a panel of chemokine-related genes, such as *CCL2, CCL3, CCL4, CCL4L2, CCL7, CCL8, CCL18, CCL20, CXCL1*, and *CXCL8*, were also upregulated in both treatment groups, indicative of immune cell recruitment and activation. Notably, HMGB1 treatment resulted in the alteration of an even broader array of cytokines, chemokines, and their receptors, including *IL23A, IL24, IL10, IL36G, IL7, IL7R, HGF, IL6ST, IL11RA, IL1RL2, IL16, IL2RA, IFITM10, IL15RA, IL18BP*, and chemokines such as *CXCL5, CXCL13, CXCL16, CXCR2, CCL22*, and *CCL23*. This highlights HMGB1’s capacity to orchestrate a more diverse and potent inflammatory cascade.

#### 3.2.2 Metallothioneins (MT1 and MT2A)

Genes from the MT1 family (*MT1G, MT1F, MT1H, MT1X, MT1E, MT1M, MT1L*) and *MT2A* were consistently upregulated in both HMGB1- and pCTS-L-treated groups. These genes are frequently associated with inflammatory responses in PBMCs and have been linked to sepsis-induced pathogenesis (37). Their induction during acute-phase inflammatory responses is a vital component of inflammation (37–40).

#### 3.2.3 S100 proteins and inflammasome components

*S100A8, S100A9*, and *S100A12*, which are DAMP molecules involved in activating immune receptors, were upregulated in both treatment groups. Critically, *CASP4* and *CASP5* were specifically upregulated only in the HMGB1-treated group. They are integral to non-canonical inflammasome pathways, leading to the maturation of inflammatory cytokines like IL-1β and IL-18 and pyroptotic cell death, particularly in response to bacterial LPS (41–43). Interestingly, consistent with our recent report (11), *Ctsl* itself was also specifically upregulated in the HMGB1-stinulated human PBMCs.

#### 3.2.4 NF-κB pathway regulators

Consistent with their pro-inflammatory properties, both mediators induced the upregulation of numerous NF-κB pathway regulators, including *NFKBIA, NFKB2, NFKB1, NFKBIZ*, and *RELB*. This highlights the central role of NF-κB signaling in mediating their pro-inflammatory effects. Additionally, *TNFAIP3* and *BCL3*, known to be associated with NF-κB signaling, were also upregulated. It is important to note that while these transcriptomic data provide strong evidence for altered pathway activity, functional validation studies will be needed to unequivocally confirm the biological activation of these pathways.

#### 3.2.5 Other important genes

Other upregulated genes in both groups included members of the TNF receptor superfamily (*TNFRSF11A, TNFRSF4, TNFSF8, TNFRSF9, TNF*) and matrix metalloproteinases (*MMP9, MMP14*), which are involved in immune cell signaling and tissue remodeling during inflammation.

### 3.3 Gene Ontology enrichment analysis

**Figure 4:**
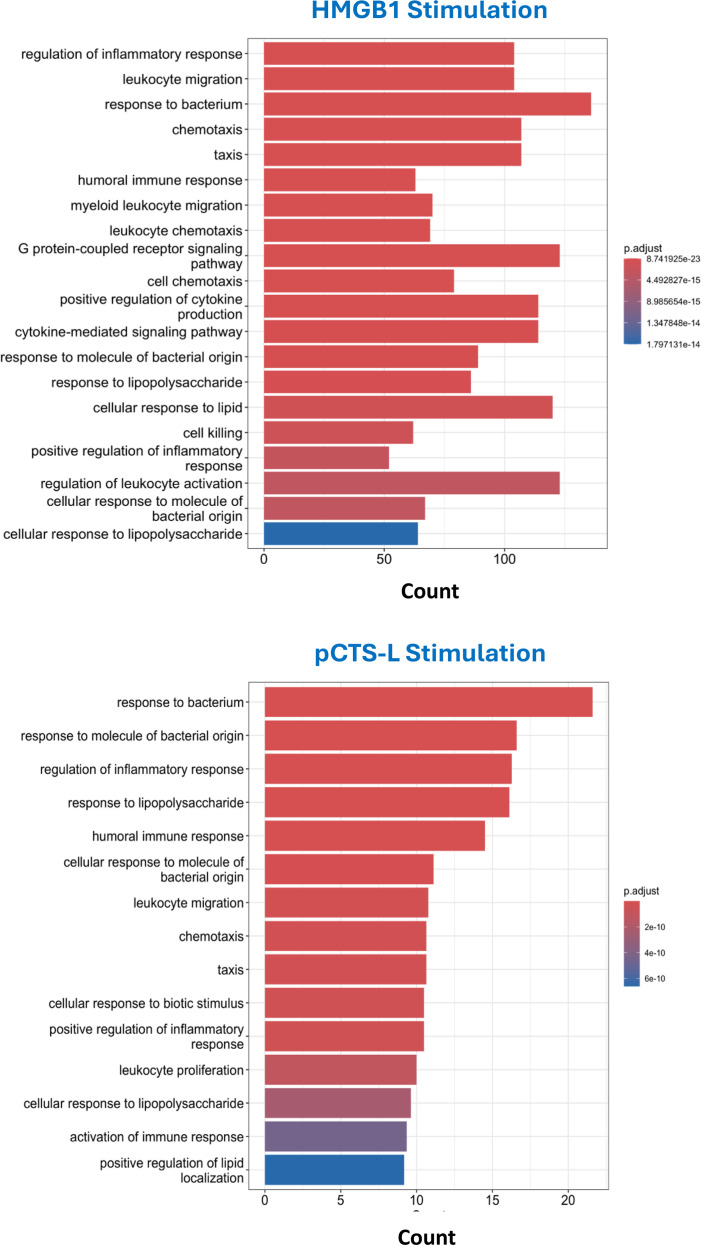
Bar plot of top 20 enriched GO biological process terms in HMGB1- or pCTSL-stimulated PBMCs. Bar graph showing the top 20 enriched GO BP terms for HMGB1- and pCTSL-induced DEGs. It depicts the enrichment scores (*e.g. P* values) and gene count as bar height.

To gain insight into the biological functions and pathways affected by HMGB1 and pCTS-L, we performed Gene Ontology (GO) enrichment analysis for biological processes on the respective DEG sets. In HMGB1-treated PBMCs, GO enrichment analysis identified “regulation of inflammatory response.” Crucially, HMGB1 treatment also significantly enriched terms related to “leukocyte migration,” “myeloid leukocyte migration,” “chemotaxis,” and “taxis” (**Figure 4**). The inclusion of migration and chemotaxis terms strongly suggests active recruitment of immune cells to sites of inflammation, characteristic of more severe inflammatory states such as sepsis and RA. In pCTSL-treated PBMCs, GO enrichment analysis similarly revealed terms such as “regulation of inflammatory response” (**Figure 4**). However, HMGB1 induces a more widespread and robust inflammatory response compared to pCTS-L.

### 3.4 Gene set enrichment analysis

**Figure 5:**
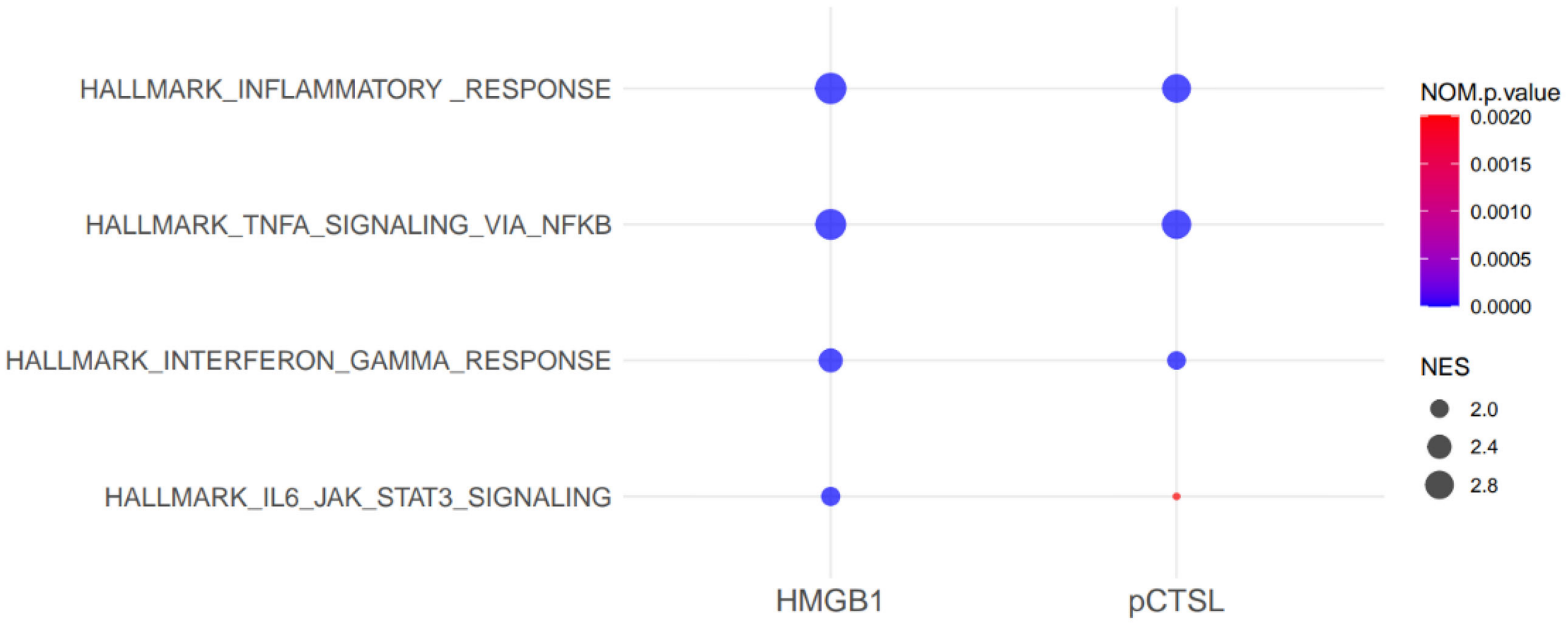
Dot plot of top 4 enriched GO biological process terms in HMGB1- or pCTSL-stimulated PBMCs. Normalized Enrichment Score (NES) accounts for differences in gene set sizes and allows comparison across gene sets. Nominal *P*-value (NOM *P*-val) indicates the statistical significance of the observed enrichment score (ES).

**Figure 6:**
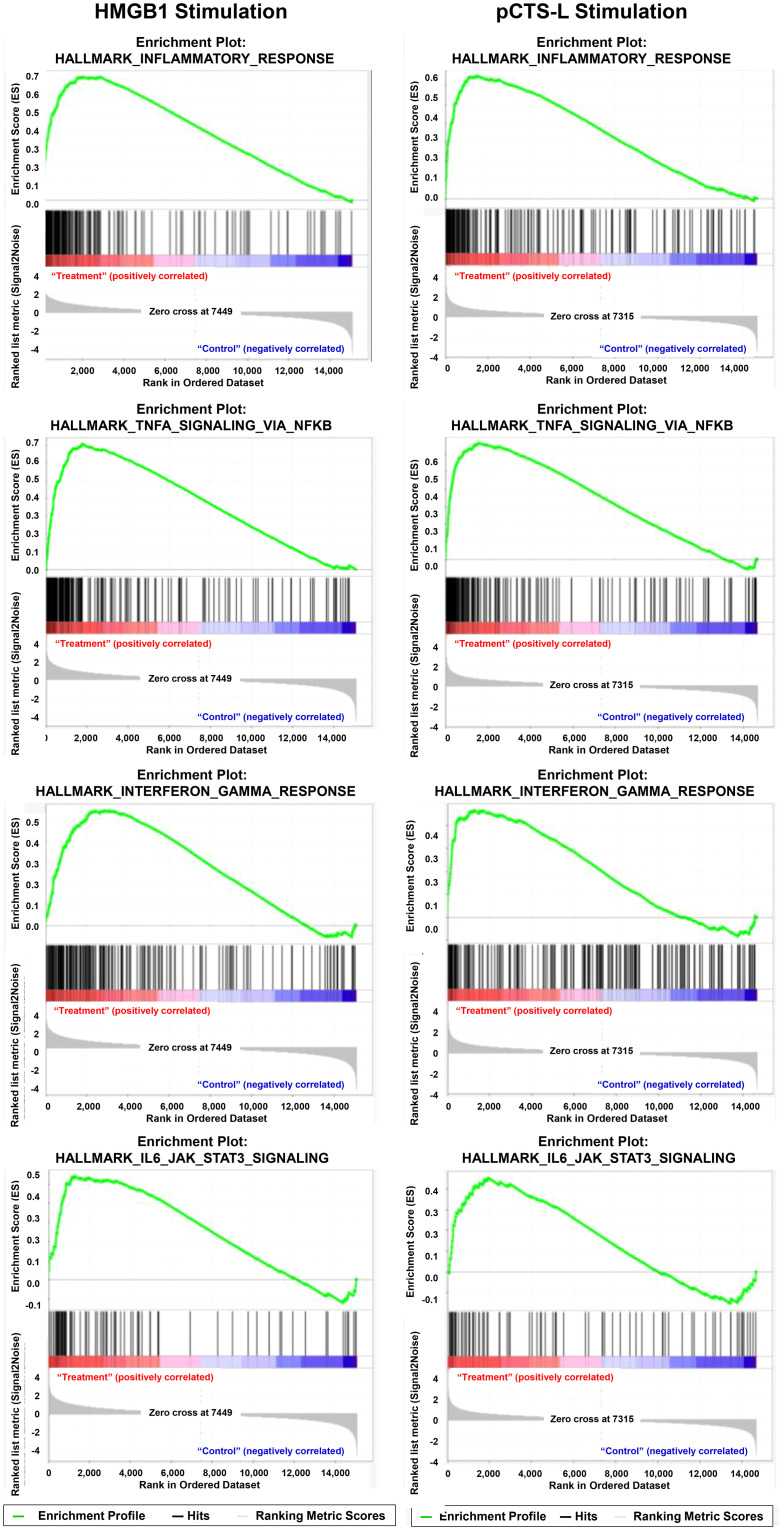
GSEA plot for hallmark signaling pathway in HMGB1- and pCTSL-stimulated PBMCs. GSEA enrichment plot or representative heatmap for top four signaling pathways in HMGB1- and pCTSL-treated cells.

Pathway analysis using GSEA with MSigDB hallmark gene sets identified a large number of inflammation-related pathways significantly altered by HMGB1 or pCTS-L, pointing toward significant immune dysregulation. Interestingly, the top four hallmark upregulated pathways were identical for both mediators (**Figures 5**, **6**): the Hallmark Inflammatory Response, the Hallmark TNF-α signaling via NF-κB, the Hallmark Interferon-gamma Response, and the Hallmark IL6-JAK-STAT3 signaling (**Figures 5**, **6**). The consistently higher Normalized Enrichment Score (NES) values for the top four hallmark pathways in the HMGB1-treated group, compared to pCTSL-treated PBMCs, further underscore the greater magnitude of inflammatory activation induced by HMGB1.

#### 3.4.1 Comparison of hallmark inflammatory response pathway

In the HMGB1-treated group, 85 genes were identified within the “Hallmark inflammatory response” pathway (**Table 3**). This extensive list encompassed nearly all genes found in the pCTS-L group (except *CMKLR1*) along with additional critical components, such as a broader range of cytokines, chemokines and receptors (*CCL5, CCL7, CCL22, IL7R, IL10, IL15RA*), more transcription factors (*IRF1, IRF7*), innate immune receptors (*TLR2, NOD2, CD14*), and cellular adhesion/migration molecules (*CD40, ITGB8, ITGB3*). This indicates a profoundly broad and amplified inflammatory response of HMGB1 stimulation.

**Table 3:** Shared genes in HMGB1- or pCTS-L-stimulated human PBMCs within four hallmark signaling pathways.

Hallmark pathways	Stimulus	Shared genes (in Bold)
Inflammatory Responses	HMGB1 (85)	ABCA1, ACVR1B, **ADM**, **ADGRE1**, **AHR**, AQP9, ATP2C1, **CCL2**, CCL5, CCL7, **CCL20**, CCL22, CCR7, CD14, CD40, CD48, CD55, **CD82**, **CHST2**, CLEC5A, CXCL6, **CXCL8**, **EBI3**, EREG, FFAR2, **FPR1**, **GCH1**, GNA15, GP1BA, GPR132, **HIF1A**, HPN, HRH1, **ICAM1**, **IL1A**, **IL1B**, **IL6**, IL7R, IL10, IL15RA, **IL18**, INHBA, **IRAK2**, IRF1, IRF7, ITGB3, **ITGB8**, LAMP3, LDLR, **LIF**, LPAR1, **LYN**, MARCO, MEFV, **MET**, **MMP14**, MXD1, **NAMPT**, NDP, **NFKB1**, **NFKBIA**, NOD2, **OSM**, P2RY2, **PDE4B**, **PDPN**, **PLAUR**, PTGER2, **PTGER4**, **PTGIR**, PVR, RASGRP1, **RGS16**, **RNF144B**, SCARF1, SCN1B, SEMA4D, SLAMF1, **SLC1A2**, SPHK1, TIMP1, TLR2, **TNFAIP6**, **TNFRSF9**, **TNFSF9**
	pCTS-L (39)	**ADM, ADGRE1, AHR, CCL2, CCL20, CD82, CHST2,** CMKLR1, **CXCL8, EBI3, FPR1, GCH1, HIF1A, ICAM1, IL1A, IL1B, IL6, IL18, IRAK2, ITGB8, LIF, LYN, MET, MMP14, NAMPT, NFKB1, NFKBIA, OSM, PDE4B, PDPN, PLAUR, PTGER4, PTGIR, RGS16, RNF144B, SLC1A2, TNFAIP6, TNFRSF9, TNFSF9**
	Both HMGB1 and pCTS-L (38)	**ADM, ADGRE1, AHR, CCL2, CCL20, CD82, CHST2, CXCL8, EBI3, FPR1, GCH1, HIF1A, ICAM1, IL1A, IL1B, IL6, IL18, IRAK2, ITGB8, LIF, LYN, MET, MMP14, NAMPT, NFKB1, NFKBIA, OSM, PDE4B, PDPN, PLAUR, PTGER4, PTGIR, RGS16, RNF144B, SLC1A2, TNFAIP6, TNFRSF9, TNFSF9**
TNF-α Signaling via NF-κB	HMGB1 (94)	ABCA1, AREG, B4GALT1, B4GALT5, **BCL3**, BCL6, BIRC3, **BTG3**, **CCL2**, **CCL4**, CCL5, **CCL20**, **CD44**, **CD80**, CHST2, CLCF1, **CXCL1**, CXCL2, CXCL3, CXCL6, DENND5A, **DRAM1**, DUSP1, DUSP4, DUSP5, **EHD1**, EFNA1, **ETS2**, **FOSL2**, FJX1, G0S2, **GCH1**, **HMGB1**, **IER3**, **IL1A**, **IL1B**, **IL6**, IL6ST, IL7R, IL15RA, **IL18**, IL23A, **ICAM1**, INHBA, IRF1, JAG1, **KYNU**, **LAMB3**, LDLR, **LIF**, LYN, **MARCKS**, MCL1, MET, MXD1, **NAMPT**, NFAT5, **NFIL3**, **NFKB1**, **NFKB2**, **NFKBIA**, NINJ1, PANX1, **PDE4B**, PFKFB3, PHLDA2, **PLAUR**, PMEPA1, PNRC1, **PTGER4**, **RELB**, **RNF19B**, SAT1, SDC4, SERPINB2, **SLC2A3**, **SLC2A6**, SLC16A6, **SOCS3**, SPHK1, TNC, TNF, **TNFAIP3**, **TNFAIP6**, **TNIP1**, TNIP2, **TNFRSF9**, **TNFSF9**, TLR2, **TRAF1**, TRIP10, **VEGFA**, **ZBTB10**, **ZC3H12A**
	pCTS-L (50)	**BCL3, BTG3, CCL2, CCL4, CCL20, CD44, CD80, CXCL1, DRAM1, EHD1, ETS2, FOSL2, GCH1, HMGB1, ICAM1, IER3, IL1A, IL1B, IL6, IL18, KYNU, LAMB3, LIF, MARCKS, NAMPT, NFKB1, NFKB2, NFKBIA, NFIL3, PDE4B,** PLAU**, PLAUR, PTGER4,** PTGS2, PTX3, **RELB, RNF19B, SLC2A3, SLC2A6, SOCS3, TNFAIP3, TNFAIP6, TNFRSF9, TNIP1, TNFSF9, TRAF1, VEGFA, ZBTB10, ZC3H12A,** CD69
	Both HMGB1 and pCTS-L (46)	**BCL3, BTG3, CCL2, CCL4, CCL20, CD44, CD80, CXCL1, DRAM1, EHD1, ETS2, FOSL2, GCH1, HMGB1, ICAM1, IER3, IL1A, IL1B, IL6, IL18, KYNU, LAMB3, LIF, MARCKS, NAMPT, NFKB1, NFKB2, NFKBIA, NFIL3, PDE4B, PLAUR, PTGER4, RELB, RNF19B, SLC2A3, SLC2A6, SOCS3, TNFAIP3, TNFAIP6, TNFRSF9, TNIP1, TNFSF9, TRAF1, VEGFA, ZBTB10, ZC3H12A**
Interferon-gamma response	HMGB1 (107)	APOL6, **ARID5B**, ARL4A, BPGM, BST2, BTG1, **C1S**, CASP1, CASP4, CASP7, **CCL2**, CCL5, CCL7, **CD38**, **CD40**, **CD274**, CDKN1A, **CFB**, **CMKLR1**, CMPK2, **CSF2RB**, DDX60, EIF4E3, EPSTI1, **FAS**, FCGR1A, **FPR1**, GBP4, **GCH1**, HELZ2, HERC6, **HIF1A**, **ICAM1**, **IDO1**, IFI27, IFI35, IFIH1, IFNAR2, IL2RB, IL4R, **IL6**, **IL7**, IL10RA, IL15RA, IRF1, IRF7, IRF9, ISG15, ISG20, **LAP3**, **LYSMD2**, **MTHFD2**, **MT2A**, **MVP**, MX1, MX2, **NAMPT**, **NFKB1**, **NFKBIA**, NLRC5, NMI, **NUP93**, OASL, OGFR, **PARP12**, **PDE4B**, **PELI1**, **PIM1**, PLA2G4A, **PLSCR1**, PML, PNP, PSMA2, PSMA3, PSMB2, PSMB9, PSME1, **PSME2**, **PTPN1**, PTPN2, **PTGS2**, RIGI, RIPK2, RSAD2, RTP4, SAMD9L, SLC25A28, **SLAMF7**, SOCS1, **SOCS3**, SPPL2A, **SRI**, SSPN, ST8SIA4, STAT1, **STAT3**, STAT4, TAP1, TAPBP, TDRD7, **TNFAIP3**, **TNFAIP6**, TRAFD1, UBE2L6, UPP1, WARS1, ZNFX1
	pCTS-L (42)	**ARID5B, C1S, CCL2,** CD69, **CD38, CD40, CD274, CFB, CMKLR1, CSF2RB, FAS, FPR1, GCH1, HIF1A, ICAM1, IDO1, IL6, IL7, LAP3, LYSMD2, MTHFD2, MT2A, MVP, NAMPT, NFKB1, NFKBIA, NUP93, PARP12, PELI1, PDE4B, PIM1, PLSCR1, PSME2, PTPN1, PTGS2, SLAMF7, SOCS3, SRI, STAT3,** TNFAIP2, **TNFAIP3, TNFAIP6**
	Both HMGB1 and pCTS-L (40)	**ARID5B, C1S, CCL2, CD38, CD40, CD274, CFB, CMKLR1, CSF2RB, FAS, FPR1, GCH1, HIF1A, ICAM1, IDO1, IL6, IL7, LAP3, LYSMD2, MTHFD2, MT2A, MVP, NAMPT, NFKB1, NFKBIA, NUP93, PARP12, PELI1, PDE4B, PIM1, PLSCR1, PSME2, PTPN1, PTGS2, SLAMF7, SOCS3, SRI, STAT3, TNFAIP3, TNFAIP6**
IL6-Jak-STAT3 response	HMGB1 (42)	ACVRL1, **ACVR1B**, **CCL7**, **CD14**, **CD38**, **CD44**, CRLF2, CSF2RA, **CSF2RB**, **CXCL1**, **CXCL3**, **EBI3**, **FAS**, **IFNAR1**, **IL1B**, **IL6**, **IL6ST**, **IL7**, **IL10RB**, **IL15RA**, IL2RA, INHBE, IRF1, ITGB3, **MAP3K8**, **PTPN1**, PTPN2, **SOCS1**, **SOCS3**, **STAT3**, **TLR2**, **TNF**
	pCTS-L (29)	**ACVR1B, CCL7, CD14, CD38, CD44, CSF2RB, CXCL1, CXCL3, EBI3, FAS,** GRB2, **IFNAR1,** IFNGR2, **IL6, IL7, IL10RB, IL15RA,** IL17RB, **IL1B, IL6ST,** LTBR, **MAP3K8,** PIM1, **PTPN1, SOCS1, SOCS3, STAT3, TLR2, TNF**
	Both HMGB1 and pCTS-L (24)	**ACVR1B, CCL7, CD14, CD38, CD44, CSF2RB, CXCL1, CXCL3, EBI3, FAS, IFNAR1, IL6, IL7, IL10RB, IL15RA, IL1B, IL6ST, MAP3K8, PTPN1, SOCS1, SOCS3, STAT3, TLR2, TNF**

The bold values highlight the genes commonly modulated by both HMGB1 and pCTS-L.

The “Hallmark inflammatory response” pathway in pCTSL-treated PBMCs showed 39 genes between the significant DEGs and the gene set (**Table 3**). These genes included inflammatory mediators such as *IL1B, IL1A, IL6, IL18, CXCL8, CCL2, CCL20*; NF-κB regulators (*NFKB1, NFKBIA*); adhesion molecules (*ICAM1*); and other inflammatory modulators (*MMP14, OSM, LIF, PDE4B, TNFRSF9, TNFSF9*).

The broad and robust upregulation of genes associated with various inflammatory response pathways in both treatment groups, but particularly accentuated in the HMGB1 group, indicates a strong and widespread activation of inflammatory programs in PBMCs. This suggests that both pCTS-L and HMGB1 elicit a significant immune response, with HMGB1 driving a more comprehensive and potentially more severe inflammatory state involving a wider network of mediators and cellular processes.

#### 3.4.2 Comparison of hallmark TNF signaling via NF-κB

In HMGB1-treated PBMCs, 94 genes were identified between the significant DEGs and those contributing to the TNF signaling via NF-κB hallmark pathway (**Table 3**). This group also included key NF-κB regulators (*NFKB1, NFKB2, RELB*), upstream receptors (*TNFRSF9, TNFSF9, CD80*), inflammatory cytokines (*IL1B, IL6, CCL2, CXCL1, IL1A, IL18, IL23A, IL15RA*), and negative feedback regulators (*TNFAIP3, NFKBIA*). Beyond the common genes with pCTS-L, HMGB1 additionally modulated expression of several chemokines (*CCL5, CCL20, CXCL2, CXCL3, CXCL6*), transcription factors and regulators (*BCL3, BCL6, IRF1, NFAT5, ZC3H12A*), receptors (*TLR2, CD44, ICAM1*), and enzymes (*PDE4B, PFKFB3, PTGER4, SOCS3*). Notably, with the exception of *PTGS2, PLAU*, and *PTX3*, all genes found in the pCTSL-associated TNF-α/NF-κB pathway were also present in the HMGB1-associated list (**Table 3**), indicating a comprehensive and amplified response by HMGB1. The consistent presence of *RELB* and *NFKB2* further supports the activation of the non-canonical NF-κB pathway.

For pCTS-L-stimulated PBMCs, a substantially smaller set of 50 genes were identified between the significant DEGs and those contributing to the TNF signaling via NF-κB hallmark pathway (**Table 3**). These included core NF-κB pathway regulators such as *NFKB1, NFKB2*, and *RELB*; upstream receptors like *TNFRSF9* and *TNFSF9*; co-stimulatory molecules like *CD80*; inflammatory cytokines (*IL1B, IL6, CCL2, CXCL1*); and negative feedback regulators (*TNFAIP3, NFKBIA*). The presence of *RELB* and *NFKB2*, but the absence of *RELA*, suggests activation of the alternate/non-canonical NF-κB pathway, which is implicated in various inflammatory diseases (44).

#### 3.4.3 Comparison of hallmark interferon signaling pathway

For HMGB1-treated PBMCs, a robust and extensive activation of the “Hallmark Interferon Signaling Pathway” was observed, with 107 co-occurring genes (**Table 3**). This signature includes key components of interferon sensing and response, such as interferon regulatory factors (IRF1, IRF7, IRF9), STAT transcription factors (STAT1, STAT3, STAT4), and interferon receptors (IFNAR2). A wide array of canonical interferon-stimulated genes (ISGs) was significantly modulated, including MX1, MX2, IFIH1, IFI35, ISG15, ISG20, OASL, and RIGI, reflecting a comprehensive antiviral and immune-modulating response. Additionally, genes involved in antigen presentation (TAP1, TAPBP) and various interferon-inducible antiviral effectors (CMPK2, DDX60, GBP4, HERC6, RSAD2, ZNFX1) were also represented. This indicates a broad and powerful induction of the interferon-mediated immune response.

In pCTS-L-stimulated PBMCs, a more constrained set of 42 genes (**Table 3**) were identified within the “Hallmark Interferon Signaling Pathway.” This group included essential interferon receptor components (IFNAR1, IFNGR2) and key downstream signaling molecules such as STAT3. Negative regulators of the pathway, SOCS1 and SOCS3, were also present, suggesting an ongoing feedback mechanism. While indicative of interferon pathway activation, the comparatively smaller number of genes, particularly the absence of many canonical ISGs and multiple IRFs seen in the HMGB1 group, suggests a more limited or perhaps an early-stage/attenuated interferon response.

Thus, both HMGB1 and pCTS-L trigger the interferon signaling pathway. However, HMGB1 induces a significantly more expansive and multifaceted interferon response, characterized by the activation of a vast array of ISGs, IRFs, and STAT proteins, alongside receptor engagement. In contrast, pCTS-L elicits a more selective interferon signature, primarily engaging key receptors and foundational signaling components. This points toward HMGB1 driving a much broader and more comprehensive interferon-driven immune activation.

#### 3.4.4 Comparison of IL-6-Jak-STAT3 signaling pathway

For HMGB1-treated PBMCs, 42 genes (**Table 3**) were identified between the significant DEGs and those contributing to the IL-6-Jak-STAT3 signaling pathway. This set included the central cytokine IL6 and the key downstream transcription factor STAT3. Also modulated were STAT3 target genes like PIM1, and important negative feedback regulators such as SOCS3 and PTPN1. This profile clearly indicates the activation of the IL-6/STAT3 axis, with mechanisms in place for its modulation.

In pCTS-L-stimulated PBMCs, 29 co-occurring genes were identified within the IL-6-Jak-STAT3 signaling pathway. This included the cytokine IL6 and a more complete representation of its receptor complex, specifically IL6ST. The central transcription factor STAT3 was also present, along with multiple negative feedback regulators: SOCS1, SOCS3, PTPN1, and PTPN2. This signature suggests a robust and well-regulated activation of the IL-6-Jak-STAT3 pathway, featuring comprehensive control mechanisms.

Thus, both HMGB1 and pCTS-L stimulate the IL-6-Jak-STAT3 signaling pathway in PBMCs. However, the pCTS-L response appears to encompass a more complete set of core pathway components, notably the IL-6 receptor subunit (IL6ST), and a broader range of negative regulators (SOCS1, PTPN2, in addition to SOCS3 and PTPN1). While both treatments activate this pathway, pCTS-L’s observed gene expression pattern might indicate a more tightly controlled or potentially a more fully developed signaling cascade within this specific pathway.

## 4 Discussion

This study utilized a comprehensive RNA-sequencing approach, coupled with Gene Ontology (GO) and Gene Set Enrichment Analysis (GSEA), to delineate the distinct and overlapping immunomodulatory effects of HMGB1 and pCTS-L on human PBMCs. Our findings reveal that while both mediators induce a significant inflammatory response, HMGB1 consistently elicits a broader and more potent transcriptional program, suggesting its role as a more comprehensive “danger signal” in inflammatory settings.

The initial analysis of differentially expressed genes (DEGs) clearly established a hierarchy in the magnitude of transcriptional change. At a relatively lower concentration, HMGB1 (0.5 µg/ml) triggered nearly four times as many DEGs (1931 genes) compared to pCTS-L (2.0 µg/ml, 489 genes). This striking difference underscores HMGB1’s capacity to orchestrate a far more expansive alteration in gene expression, impacting a wider array of cellular processes. Despite this quantitative difference, the identification of 412 commonly regulated DEGs between HMGB1 and pCTS-L, however, confirms the existence of shared inflammatory mechanisms. These involve the upregulation of core pro-inflammatory cytokines (*IL1A*, *IL1B*, *IL6*, and *OSM*), interferons (*IFNA*, *IFNB*, and *IFNG*) and chemokines (*CCL2*, *CCL3*, *CCL4*, *CCL7*, *CXCL1*, and *CXCL8*), consistent with their roles as proinflammatory mediators in initiating immune cell activation and recruitment. The upregulation of metallothionein genes (*MT1, MT2A*) in both groups further supports a general inflammatory response, which is a common feature in conditions like sepsis (37–40). Similarly, the shared upregulation of S100 proteins (*S100A8, S100A9*, and *S100A12*), known DAMPs involved in activating immune receptors in response to viral infections (45), highlights a common mechanism of amplifying inflammatory signaling by both HMGB1 and pCTS-L.

However, HMGB1’s superior capacity to induce inflammation was evident in several specific aspects. The HMGB1-stimulated group uniquely showed upregulation of *CASP4* and *CASP5* (functional homologs of mouse *CASP11*), which are critical for the non-canonical inflammasome pathway that triggers pyroptotic cell death and the maturation of inflammasome-dependent cytokines (e.g., IL-1β and IL-18) (41–43). This finding was consistent with our earlier report that HMGB1 mediates CASP-11-dependent pyroptosis in sepsis (13). This suggests that HMGB1 might directly trigger or significantly contribute to inflammasome activation, a hallmark of severe inflammatory responses often seen in sepsis. In peritoneal macrophages derived from wildtype but not from Tlr4/Rage-deficient mice, pCTS-L similarly induced pro-Casp-11 expression and CASP-11 maturation, suggesting a possible role for pCTS-L in CASP-11-associated pyroptosis and immunosuppression (25). However, the absence of CASP4/CASP5 upregulation in the pCTS-L group suggests that HMGB1 engages a more severe, potentially cell-damaging, form of inflammatory response in many innate immune cell types. Interestingly, cathepsin L (*Ctsl*) itself was also specifically upregulated by HMGB1 (11), suggesting potential feedback loops or cross-talk between these two inflammatory mediators. Moreover, HMGB1 modulated a larger and more diverse set of cytokines and chemokines, including *IL23A, IL24, IL10, CXCL5, CXCL13*, and *CCL22*, implying a broader range of immune cell activation and recruitment mechanisms. Notably, our current study aligns with previous research demonstrating that HMGB1 downregulates IL24 expression in other cell types, specifically keratinocytes (46).

Gene Ontology enrichment analysis provided functional insights into these transcriptional changes. Both mediators activated terms related to bacterial response, general inflammatory regulation, chemotaxis, and leukocyte migration. These findings are critical because they suggest that both HMGB1 and pCTS-L not only induce local inflammation but also actively promote the movement and recruitment of immune cells, characteristic of systemic inflammatory responses like those seen in sepsis, where uncontrolled leukocyte infiltration can lead to tissue damage.

The GSEA, utilizing MSigDB hallmark gene sets, corroborated and expanded upon these findings. The observation that the top four upregulated hallmark pathways were identical for both mediators—Hallmark Inflammatory Response, Hallmark TNF-α signaling via NF-κB, Hallmark Interferon-gamma Response, and Hallmark IL6-JAK-STAT3 signaling—highlights the common central axes of immune activation. However, HMGB1 consistently yielded higher Normalized Enrichment Scores (NES) for these pathways and involved a substantially greater number of genes within them (e.g., 92 genes in TNF/NF-κB for HMGB1 vs. 49 for pCTS-L). Our findings align with a recent report demonstrating that HMGB1, through its receptor-ligand activity (e.g., cytokine receptor binding), activates multiple signaling pathways in dendritic cells—including NF-κB and Jak–STAT—thereby orchestrating immune regulation (47). This quantitative difference highlights HMGB1’s ability to drive a more intense and broadly distributed activation of these critical inflammatory circuits.

Delving into specific hallmark pathways revealed further nuances. In the Hallmark Inflammatory Response, HMGB1 activated a significantly broader set of 85 genes, encompassing nearly all those induced by pCTS-L, but adding a wider spectrum of cytokines/chemokines (e.g., CCL5, CCL7, CCL22, IL10, IL15RA), transcription factors (IRF1, IRF7), and key innate immune receptors (TLR2, NOD2, CD14, CD40). This comprehensive activation positions HMGB1 as a master regulator of the inflammatory cascade, capable of initiating a highly complex and amplified response.

A consistent mechanistic thread emerging from our analysis is the activation of the non-canonical NF-κB pathway, evidenced by the upregulation of *NFKB2* and *RELB* in both HMGB1 and pCTS-L-stimulated cells. Our findings are consistent with previous reports that HMGB1 stimulates leukocyte migration through the IKKα-dependent NF-κB p52/RelB noncanonical pathway (44). The non-canonical NF-κB pathway, also known as the alternative NF-κB pathway, is one of two major signaling cascades that lead to the activation of the Nuclear Factor kappa-light-chain-enhancer of activated B cells (NF-κB) transcription factor family (48). Unlike the canonical pathway, which is rapid and primarily involved in immediate inflammatory responses, the non-canonical pathway is generally slower, more sustained, and plays a crucial role in specific developmental processes and adaptive immune responses (49). Its dysregulation has been linked to various inflammatory and autoimmune diseases, including sepsis and RA (50–52). The engagement of these inflammatory pathways by both mediators suggests a common but potentially nuanced mechanism through which they exert their immunomodulatory effects, warranting further investigation into its precise contribution to HMGB1- and pCTS-L-mediated inflammation. Although these transcriptomic data provide strong evidence for altered pathway activity, functional validation studies would be crucial to unequivocally confirm the biological activation of these pathways.

The Hallmark Interferon-gamma Response showed the most dramatic disparity. HMGB1 activated a robust and extensive signature of 107 genes, including multiple *IRFs* (*IRF1, IRF7*, *IRF9*), STAT transcription factors (*STAT1*, *STAT3*, *STAT4*), interferon receptors (*IFNAR2*), and a vast array of canonical interferon-stimulated genes (ISGs) such as *MX1*, *MX2*, *IFIH1*, *ISG15*, *ISG20*, *OASL*, and *RIGI*. This comprehensive induction signifies HMGB1’s powerful role in orchestrating a broad antiviral and immunomodulatory response, often characteristic of severe inflammatory and infectious states. In contrast, pCTS-L induced a more constrained set of 42 genes, primarily including key interferon receptor components (*IFNAR1*, *IFNGR2*) and foundational signaling molecules like STAT3, along with negative regulators (*SOCS1*, *SOCS3*). This suggests a more selective or attenuated interferon response by pCTS-L, lacking the widespread *ISG* and *IRF* induction seen with HMGB1. Thus, both HMGB1 and pCTS-L trigger the interferon signaling pathway. However, HMGB1 induces a significantly more expansive and multifaceted interferon response, characterized by the activation of a vast array of ISGs, IRFs, and STAT proteins, alongside receptor engagement. In contrast, pCTS-L elicits a more selective interferon signature, primarily engaging key receptors and foundational signaling components. This points toward HMGB1 driving a much broader and more comprehensive interferon-driven immune activation.

Interestingly, the IL-6-Jak-STAT3 Signaling Pathway presented a unique pattern. While both HMGB1 (42 genes) and pCTS-L (29 genes) activated this pathway, the pCTS-L response appeared to encompass a more complete set of core pathway components, notably the IL-6 receptor subunit (*IL6ST*) and a broader range of negative feedback regulators (*SOCS1*, *SOCS3*, *PTPN1*, and *PTPN2*). This intriguing finding suggests that while HMGB1 may drive a larger overall inflammatory output, the IL-6-Jak-STAT3 pathway, a critical regulator of immune homeostasis and inflammation resolution, might be more robustly or comprehensively controlled in response to pCTS-L. This could imply a more nuanced or finely tuned immunomodulatory role for pCTS-L within this specific signaling axis, potentially mediating specific aspects of tissue repair or resolution, rather than solely promoting broad-spectrum inflammation.

These findings have significant implications for understanding the pathogenesis of inflammatory diseases, particularly those involving DAMPs like HMGB1 and inducible cytokines like pCTS-L. HMGB1’s capacity to induce a broader, more intense inflammatory and interferon response, coupled with the activation of non-canonical inflammasome components and active leukocyte migration, aligns with its established role as a critical mediator of sepsis, sterile inflammation, and autoimmune diseases. The observation that HMGB1 and pCTS-L share significant core inflammatory pathways but differ in the breadth and specific features of their induced responses highlights the complexity of DAMP-mediated immunity. Understanding these distinctions is crucial for the development of targeted therapies. For instance, interventions aimed at HMGB1 might need to address a wider array of downstream inflammatory and migratory processes, whereas pCTS-L inhibition might modulate a more constrained set of responses. The “cross-disease” approach of investigating sepsis mediators in other inflammatory diseases (such as RA) is strongly supported by these results, as the fundamental inflammatory and interferon signaling pathways triggered by HMGB1 and pCTS-L are common to many pathologies.

This study provides a comprehensive transcriptomic snapshot of PBMCs stimulated by HMGB1 and pCTS-L *in vitro*. However, it has certain limitations. Although demographic or clinical information, such as age, sex, race, or smoking status, can influence gene expression and cellular responses to various stimuli, we were not provided with these detailed demographic or clinical information for the individuals who donated blood for PBMC isolation. The *in vitro* nature of the human PBMC model, while providing a controlled environment, does not fully recapitulate the complex cellular interactions and systemic milieu present *in vivo*. Our analysis was conducted at a single time point (16 hours), and inflammatory responses are highly dynamic; a time-course study could reveal transient or delayed effects. Furthermore, bulk RNA sequencing provides an average transcriptional profile across the entire PBMC population. Given that PBMCs comprise various cell types (monocytes, T cells, B cells, NK cells), single-cell RNA sequencing would offer a more granular understanding of cell-type specific responses to HMGB1 and pCTS-L. Finally, while RNA-seq identifies transcriptional changes, validation at the protein level (e.g., cytokine secretion, NF-κB subunit activation) would further strengthen these findings. Investigating the upstream receptor-ligand interactions that drive these differential responses, especially for the CASP4/CASP5 and the distinct IL-6-Jak-STAT3 pathway regulation, would be valuable. Finally, exploring these mechanisms in *in vivo* models of sepsis or autoimmune diseases would be crucial to translate these *in vitro* findings into a physiological context.

Despite these limitations, our study significantly advances the understanding of HMGB1 and pCTS-L as inflammatory mediators. The direct comparison highlights both their shared inflammatory triggers and the distinctly broader and potentially more severe transcriptional program orchestrated by HMGB1. These insights are crucial for understanding the pathogenesis of HMGB1- and pCTS-L-driven inflammatory diseases and for the development of targeted therapeutic strategies. For instance, interventions against HMGB1 might be particularly effective in conditions characterized by widespread immune cell activation and migration, such as severe sepsis, while targeting pCTSL could address more specific inflammatory components.

In conclusion, our RNA-sequencing based comparative study reveals that both HMGB1 and pCTS-L are potent immunomodulatory mediators in human PBMCs. While both induce robust inflammatory gene expression profiles, HMGB1 elicits a significantly more extensive and diversified transcriptional response, characterized by a greater number of differentially expressed genes, unique activation of inflammasome components (CASP4, CASP5), a broader spectrum of cytokines and chemokines, and pronounced pathways related to leukocyte migration and chemotaxis. Both mediators converge on the activation of the non-canonical NF-κB pathway. These findings underscore the distinct and overlapping roles of HMGB1 and pCTS-L in orchestrating immune responses, providing valuable insights for deciphering the complexities of HMGB1/pCTS-L-mediated inflammation and informing the development of targeted therapeutic strategies for inflammatory diseases.

## Data Availability

The data presented in the study are deposited in the NCBI repository, accession number PRJNA1390761 (https://www.ncbi.nlm.nih.gov/sra/PRJNA1390761).
